# Clioquinol inactivates thiamine pyrophosphate by increasing cellular oxidative stress

**DOI:** 10.1016/j.redox.2026.104258

**Published:** 2026-06-17

**Authors:** Zheyu Fan, Xianghui Yan, Qiaoqiao Zheng, Ziting Zhu, Boyi Song, Xiao Feng, Siyu Pan, Chunyi Lv, Ping Shi

**Affiliations:** State Key Laboratory of Bioreactor Engineering, East China University of Science and Technology, Shanghai, 200237, China

**Keywords:** Clioquinol, Neurotoxicity, Oxidative stress, Thiamine deficiency, Metabolic reprogramming, Mitochondrial dysfunction

## Abstract

Clioquinol (CQ), a prescribed oral antibiotic, was withdrawn from use following its association with the incidence of subacute myelo-optic neuropathy (SMON) in Japan. The neurotoxicity associated with CQ may be linked to thiamine deficiency. However, their relationship and underlying mechanisms remain unclear. In this study, we identified thiamine pyrophosphate (TPP) as a key metabolite affected by CQ via metabolomics analysis. Analysis of related thiamine transport, TPP synthesis, and oxidative stress pathways revealed that CQ promoted TPP inactivation through oxidative modification. The Seahorse metabolic analyzer data showed that CQ-induced TPP deficiency led to diminished mitochondrial oxidative phosphorylation, accompanied by enhanced glycolysis. Morphological examination of mitochondria further indicated that CQ elicited mitochondrial damage in a TPP-dependent manner. In an Alzheimer's disease murine model, CQ administration similarly provoked oxidative stress, decreased cerebral TPP concentrations, and induced neuronal injury. Notably, supplementation with TPP or N-acetylcysteine (NAC) mitigated CQ-associated neurotoxicity and potentiated CQ-mediated clearance of amyloid plaques. Our findings elucidate that CQ is involved in mitochondrial dysfunction and metabolic reprogramming by inactivating TPP, providing valuable insights into the neurotoxicity of CQ.

## 1 Introduction

Clioquinol (5-chloro-8-hydroxy-7-iodoquinoline, CQ) is a halogenated 8-hydroxyquinoline that served as an oral antiparasitic agent for the prevention and treatment of intestinal amoebiasis from the 1950s until the 1970s. However, in the 1970s, the oral formulation of CQ was withdrawn from the market due to concerns regarding its association with SMON. Additionally, CQ functions as a metal chelator and is posited to reduce amyloid levels via copper-dependent mechanisms in Alzheimer's disease (AD) pathology models, thereby being considered a potential therapeutic agent for AD [1]. Clinical trials have demonstrated that CQ can reduce plasma levels of amyloid beta 42 (Aβ42) in patients [2]. However, a subsequent phase II clinical study indicated that CQ did not significantly enhance cognitive function and was associated with neurological symptoms, including impairments in visual and color perception, which may be linked to the neurotoxicity of CQ [3]. Our previous studies have demonstrated that CQ inhibits the proliferation of SH-SY5Y cells by inducing S-phase cell cycle arrest through the regulation of calcium ions mediated by calreticulin (CRT) and aggravates autophagy via the CRT-mediated unfolded protein response (UPR) pathway in human neurotypic SH-SY5Y cells [4].

Thiamine, or vitamin B1, is an essential water-soluble vitamin necessary for mitochondrial energy metabolism. Its active form, TPP, functions as a coenzyme for critical enzymes involved in glucose, fatty acid, and amino acid metabolic pathways [5], including pyruvate dehydrogenase, α-ketoglutarate dehydrogenase, and transketolase [6]. Clinical manifestations of thiamine deficiency can include severe lactic acidosis and primary neurological disorders [7,8]. The quinoline derivative chloroquine was reported to inhibit thiamine uptake by downregulating the thiamine transport protein in both yeast and human cell lines [9]. Herein, we hypothesized that the neurotoxicity of CQ may similarly be related to thiamine levels through an analogous mechanism of chloroquine.

In this study, we investigated the effects of CQ on the concentrations of thiamine and its derivatives in animal cells and examined the relationship between the toxicity of CQ and these observed effects. Subsequently, we explored the underlying mechanisms through which CQ exerts these effects. Additionally, we assessed the effects of CQ on thiamine and its derivatives in both brain tissue and serum in AD mice and evaluated potential combination therapy strategies to mitigate CQ-associated toxicity. Our results revealed that CQ induces oxidative stress, leading to the inactivation of TPP. This inactivation further exacerbates thiamine depletion, thereby contributing to mitochondrial dysfunction and metabolic reprogramming. The findings enhance our understanding of CQ's neurotoxicity and provide a theoretical framework for the development of CQ derivatives for the treatment of AD.

## 2 Materials and methods

### 2.1 Cell lines and culture conditions

The human neuroblastoma cell line SH-SY5Y and the human renal epithelial cell line HEK293T were obtained from Cell bank of Chinese Academy of Sciences. HEK293T and SH-SY5Y cell lines were cultured in DMEM/high glucose medium with 10% FBS and 1% penicillin/streptomycin. All cell lines were incubated at 37 °C in a humidified atmosphere with 5% CO_2_.

### 2.2 Animals

APP/PS1 model mice and WT mice (C57BL/6 background, six weeks old) were provided by Aniphe Biolaboratory Inc. The mice were housed in a specific pathogen-free environment at 25 °C with a 12-h light/12-h dark cycle. During the experiment, animals were provided free access to food and water. The APP/PS1 model mice and WT mice were genotyped by PCR. All animal experiments were approved by the ECUST Animal Management and Ethics Committee (Approval No: ECUST-2025-016).

### 2.3 Animal administration

After adapting to the new environment for one week, the mice were randomly divided into four groups: the control group (n = 6/group) was given a gavage of solvent (0.5% carboxymethyl cellulose, 5% DMSO, 0.9% sodium chloride) at a dose of 15 mL/kg/d; the CQ treatment group (n = 6/group) received a gavage of 90 mg/kg/d CQ; the CQ + NAC treatment group (n = 6/group) received a gavage of 90 mg/kg/d CQ and 150 mg/kg/d NAC; and the CQ + TPP group (n = 6/group) received a gavage of 90 mg/kg/d CQ and 150 mg/kg/d TPP, with all drugs prepared in a solution of 0.5% carboxymethyl cellulose, 5% DMSO, and 0.9% sodium chloride. The doses of NAC and TPP were based on studies related to neuroprotection [[10], [11], [12], [13], [14], [15], [16]]. After continuous gavage for 30 days, the mice were anesthetized, their eyes were removed for blood collection, and then they were euthanized by spinal dislocation, and the brains were collected.

### 2.4 Animal sample collection

Blood samples were obtained through the orbit, and the animals were euthanized using the spinal dislocation method. The brains of the mice were quickly collected and divided into two halves. One half was preserved in 4% paraformaldehyde, while the other half was rapidly frozen in liquid nitrogen and then stored at −80 °C. After allowing the blood samples to sit at room temperature for 1 h, they were centrifuged at 3000 rpm for 10 min. The supernatant was collected and centrifuged again at 3000 rpm for 5 min, after which the supernatant was taken and stored at −80 °C.

### 2.5 Cell viability determination

The cell viability assay was performed using the CCK-8 assay (APExBIO, Houston, USA). Cells were cultured in 96-well plates (7000 cells/well) and incubated overnight at 37 °C in 5% CO_2_. The cells were then treated with the target compounds for 24 h. Cell viability was then determined by the CCK-8 assay, performed in strict accordance with the manufacturer's instructions. The IC50 value was defined as the concentration that caused 50% loss of cell viability and was calculated using GraphPad Prism 8.

### 2.6 High-throughput targeted metabolomics by UHPLC-MS analysis

The metabolites were extracted from cell residue with 1 mL precooled methanol/acetonitrile/water (v/v, 2:2:1) under sonication for 1 h in ice baths. The mixture was incubated at −20 °C for 1 h, followed by centrifugation at 16000g, 4 °C for 20 min, and then transferred to the sampling vial for LC-MS analysis. The LC/MS portion of the platform was based on a Shimadzu Nexera X2 LC-30AD system equipped with an ACQUITY UPLC HSS T3 column (1.7 μm, 2.1 mm × 100 mm, Waters) and a triple quadrupole mass spectrometer (5500 QTRAP, AB SCIEX). Metabolites were detected in electrospray negative-ionization and positive-ionization modes. Widely targeted metabolites were quantified using multiple-reaction monitoring mode set up as previously reported [17,18].

MultiQuant 3.0.2 software was used to extract the original MRM data of QMT1000 KIT metabolites and obtain the peak area of each metabolite for quantification from different samples. The discriminating metabolites were obtained using a statistically significant threshold of variable influence on projection (VIP) values obtained from the OPLS-DA model and a two-tailed Student's *t*-test (p-value) on the peak area of each metabolite for comparison between the two groups. The p-value was calculated by one-way analysis of variance (ANOVA) for multiple groups analysis. Metabolites with VIP > 1 and P-value< 0.05 were considered to be statistically significant. The significantly differential metabolites were used to perform cluster analyses with the R package. To identify the perturbed biological pathways, the differential metabolite data were subjected to KEGG pathway analysis using the KEGG database (http://kegg.jp). KEGG enrichment analyses were carried out with the Fisher's exact test, and FDR correction for multiple testing was also performed.

### 2.7 Detection of thiamine derivation and oxidized thiamine derivation by HPLC

A thiochrome-based HPLC method was used to measure thiamine and its derivatives, including TPP, in cells, serum, and tissues, with a limit of quantification (LOQ) as low as 3 nmol/L [[19], [20], [21], [22]]. The HPLC system was an Agilent 1100 series equipped with a degasser, quaternary pump, and fluorescence detector. Separation was performed at 25 °C using an Eclipse XDB-C18 (Agilent, California, USA). The mobile phase A was 25 mM Na_2_HPO_4_ in water (pH 7.4), and the mobile phase B was 100% methanol. Gradient steps were as follows: 10-20% B in 3 min, 20-40% B in 5 min, held at 40% B for 5 min, returned to initial conditions (5% B) in 2 min, and equilibrated for 5 min (total run time 20 min). The flow rate and injection volume were 0.8 mL/min and 10 μL, respectively. Thiochrome compounds were detected at an excitation wavelength of 375 nm and an emission wavelength of 435 nm. The standard curves used for detecting thiamine and TPP are shown in the supplementary file (Fig. S1).

### 2.8 RT-qPCR

RNA extraction was performed using Total RNA Extraction Reagent (YEASEN, Shanghai, China), and cDNA synthesis was carried out using Hifair Ⅲ 1st Strand cDNA Synthesis SuperMix for qPCR (YEASEN, Shanghai, China). RT-qPCR was conducted using Hifair qPCR SYBR Green Master Mix (YEASEN, Shanghai, China) and specific primers. All the above experiments were strictly conducted according to the instructions. 18S and HPRT were used as housekeeping genes, and the relative change levels of the target gene were calculated using the 2^−ΔΔCt^ method. Primers sequences were listed in Table S1.

### 2.9 Western Blotting

Cell lysis and protein extraction were performed as described [4]. The total protein concentration in the lysates was measured using the Coomassie brilliant blue determination method. After separating using SDS-PAGE, proteins were then transferred to PVDF membranes (Cytiva, Marlborough, USA). After sealing with 5% skim milk (prepared in TBST) at room temperature for 2 h, incubate with the primary antibody (Proteintech, Wuhan, China) overnight at 4 °C. Then incubate with the secondary antibody (Epizyme, Shanghai, China) at room temperature for 2 h. After each incubation, wash the membrane three times with TBST, 5 min each time. Use the Tanon system to capture Western Blotting images and analyze band intensity using ImageJ.

### 2.10 TPK1 enzyme activity assay

To determine the TPK activity, the TPK1 protein was recombinantly expressed using pET28a and purified through nickel column chromatography to obtain pure protein. The reaction system consisted of 50 mM Tris HCl, 40 μM MgSO_4_, 40 μM ATP, and 2 μg/mL TPK1. The reaction was initiated by adding thiamine to a final concentration of 100 μM and incubated at 37 °C for 1 h. The reaction was terminated by adding trichloroacetic acid (10%). The mixture was centrifuged at 10000 g for 15 min at 4 °C to precipitate the protein, and then the levels of TPP and thiamine in the supernatant were measured by HPLC.

To assess the enzymatic activity of intracellular TPK1, crude TPK1 enzyme was extracted from SH-SY5Y cells [23]. The cells exhibiting normal growth were harvested and washed twice with pre-chilled PBS. Following the removal of the supernatant, the cells were lysed using a cell lysis buffer (50 mM Tris-HCl, pH 7.5; 150 mM NaCl; 1 mM EDTA; 0.5% Triton X-100) containing protease inhibitors. The lysates were then centrifuged at 12000 g for 10 min at 4 °C, and the resulting supernatant was collected as the crude enzyme extract. An appropriate amount of crude enzyme (containing 50–500 μg of protein) was added to the reaction mixture composed of 50 mM Tris-HCl (pH 7.5), 5 mM MgCl_2_, 1 mM ATP, and 0.1 mM thiamine. The volume was adjusted to 200 μL with deionized water. After incubation at 37 °C for 15 to 30 min, the reaction was terminated by adding 100 μL of 50% TCA. Subsequent sample processing was conducted following the established protocol for cellular thiamine extraction. TPK activity is expressed as the amount of TPP produced (nmol) per minute per milligram of protein.

### 2.11 ROS detection

Seeded SH-SY5Y cells in a 6-well plate at a density of 3.6 × 10^6^ cells per well and incubated overnight at 37 °C. Then, treat with fresh medium containing the target drug for 24 h. Cells were incubated with DCFH-DA (Beyotime, Shanghai, China) for 30 min in the dark following exposure to the target drug for 24 h, respectively. Fluorescence was analyzed by flow cytometry.

### 2.12 Total GSH, MDA, SOD and CAT enzyme activities assay

Treated SH-SY5Y cells with the target drug for 24 h. After treatment, measure MDA (Njjcbio, Nanjing, China), GSH (Beyotime, Shanghai, China), SOD (Beyotime, Shanghai, China), and CAT (Beyotime, Shanghai, China) enzyme activity according to the instructions in the kit.

### 2.13 Detection of thiochrome and thiamine in medium

Collected the cell culture medium after drug treatment, centrifuge at 12000 rpm for 10 min, take 200 μL of the supernatant as the sample or standard, add 50 μL of methanol, mix well, then add 125 μL of freshly prepared 0.3 mM K_3_Fe(CN)_6_, mix well, and then detect the emission signal at 375 nm and excitation signal at 435 nm.

### 2.14 Mitochondrial membrane potential detection

SH-SY5Y cells were seeded in a 6-well plate at a density of 3.6 × 10^6^ cells per well and cultured overnight in an environment with 5% CO_2_ at 37 °C. The cells were then treated with the target drug for 24 h. Subsequently, detection was performed according to the JC-1 (YEASEN, Shanghai, China) instruction manual.

### 2.15 ATP assay

SH-SY5Y cells were seeded in a 6-well plate at a concentration of 3.6 × 10^6^ cells per well and cultured overnight in an environment containing 5% CO_2_ at 37 °C. The cells were then treated with the target drug for 24 h. ATP levels in cells were measured using an enhanced ATP detection kit (Beyotime, Shanghai, China) according to the instructions, and relative quantification was normalized to total protein.

### 2.16 Energy metabolism analysis

The Cell Mito Stress Test kit (Agilent, California, USA) was used to measure cellular oxidative phosphorylation levels. The Glycolysis Stress Test kit (Agilent, California, USA) was used to assess cellular glycolysis levels. Measurements were performed using the XF96 Extracellular Flux Analyzer according to the manufacturer's instructions. SH-SY5Y cells were seeded at a density of 6000 cells per well in an XF96 cell culture microplate and then incubated overnight at 37 °C in a 5% CO_2_ incubator. To measure glycolysis levels, 0.5 μM rotenone/antimycin and 50 mM 2-DG were injected into each well. To measure cellular oxidative phosphorylation levels, 1.5 μM oligomycin, 0.5 μM FCCP, and 0.5 μM rotenone/antimycin were sequentially injected into each well. All Seahorse data were normalized to protein, with all metabolic parameters automatically calculated by the WAVE software equipped in the Seahorse.

### 2.17 Pathological morphology staining, IHC, and IF

Nissl staining was employed to assess neuronal damage in brain tissue. Immunohistochemical analysis targeting Aβ42 was performed to assess amyloid plaque clearance. Additionally, immunofluorescence staining for 4-HNE was used to assess oxidative stress in the brain tissue. All procedures involving Nissl staining, immunohistochemistry, and immunofluorescence were performed following standardized experimental protocols.

### 2.18 Statistical analysis

Values are expressed as mean ± SD. Statistical analysis was performed using Student's t-test. For multiple treatment groups, a two-way analysis of variance was applied. All analyses were performed using GraphPad Prism8. Significant differences were determined by P < 0.05.

## 3 Results

### 3.1 CQ reduces TPP levels in SH-SY5Y cells

**Fig. 1:**
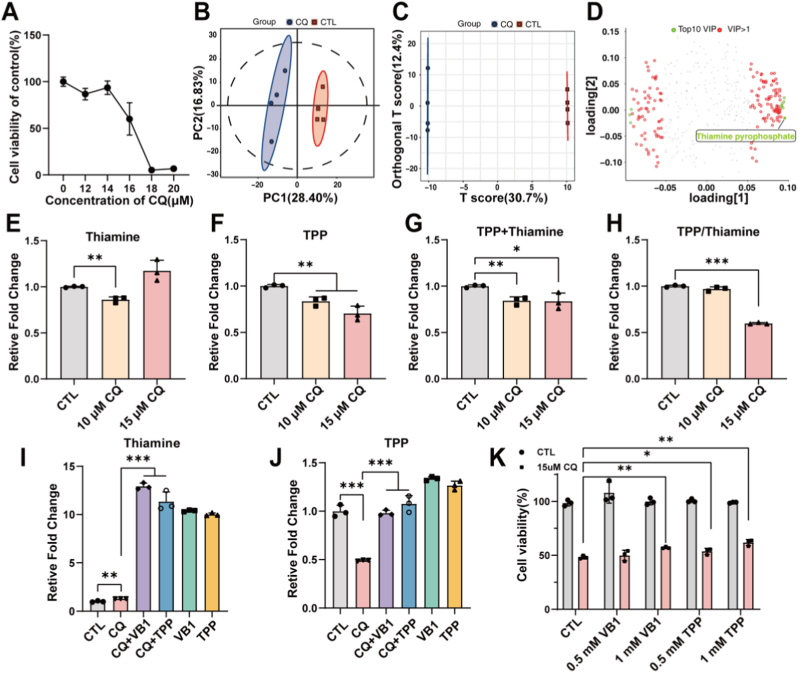
The effect of CQ on intracellular thiamine and TPP in SH-SY5Y cells. (A) The CCK-8 method was used to determine the effect of different concentrations of CQ (12, 14, 16, 18, 20 μM) treatment for 24 h on the proliferation of SH-SY5Y cells. (B) Principal component analysis (PCA) plot of high-throughput targeted metabolomics. (C) Orthogonal partial least squares discriminant analysis (OPLS-DA) plot of high-throughput targeted metabolomics. (D) Load diagram of OPLS-DA. Red indicates metabolites with VIP greater than 1, and green indicates the top ten metabolites with the highest VIP. TPP is labeled by a solid green circle in the diagram. (B-D) Data was detected by LC-MS/MS after treatment with 15 μM CQ in SH-SY5Y (n = 4). (E) The intracellular levels of thiamine, (F) The intracellular levels of TPP. (G) The relative level of the sum of thiamine and TPP within the cell. (H) The relative level of the ratio of TPP to thiamine within the cell. (E-H) The data were detected by HPLC after 24 h of CQ at different concentrations in SH-SY5Y. (I and J)The levels of intracellular thiamine and TPP in SH-SY5Y cells were detected by HPLC in the groups of CTL, CQ, CQ + VB_1_, CQ + TPP, VB_1_, and TPP, where the concentration of CQ is 15 μM, and the concentrations of VB_1_ and TPP are both 1 mM. (K) The bar chart shows the alleviation of cytotoxicity of 15 μM CQ in the CTL, 0.5 mM VB_1,_ 1 mM VB_1_, 0.5 mM TPP, and 1 mM TPP groups. Data (n = 3) were presented as mean ± SE. ∗, P < 0.05; ∗∗, P < 0.01; ∗∗∗, P < 0.001.

We initially evaluated the IC50 of CQ in SH-SY5Y cells utilizing the CCK-8 assay. As illustrated in Fig. 1A, the IC50 of CQ in SHSY5Y cells was approximately 15 μM. To elucidate the metabolic alterations induced by CQ, SH-SY5Y cells were exposed to 15 μM CQ for 24 h, followed by analysis via high-throughput targeted metabolomics. Principal component analysis (PCA) and orthogonal partial least squares discriminant analysis (OPLS-DA) distinctly segregated the samples into two groups, reflecting divergent metabolic profiles (Fig. 1B and C). Variable importance in projection (VIP) scores derived from OPLS-DA were computed to evaluate the contribution and explanatory capacity of each metabolite's expression pattern in discriminating between sample groups, thereby facilitating the identification of potential biomarker metabolites. In the OPLS-DA loading plot, the top ten metabolites ranked by VIP scores were indicated with green circles, whereas other metabolites exhibiting VIP values exceeding 1 were denoted with red circles (Fig. 1D). Among these top differential metabolites, TPP, the active form of thiamine and an essential coenzyme in cellular energy metabolism, attracted our attention (a green solid circle, Fig. 1D).

Then, SH-SY5Y cells were subjected to treatment with 10 μM and 15 μM CQ for a duration of 24 h, after which the intracellular levels of thiamine and its derivatives were quantified using HPLC [[19], [20], [21], [22]]. The findings indicated that treatment with 10 μM CQ resulted in a significant reduction in thiamine and TPP levels (Fig. 1E and F). In contrast, treatment with 15 μM CQ significantly decreased only TPP levels (Fig. 1E and F). It was important to note that under 15 μM CQ treatment, both the total intracellular thiamine level (thiamine + TPP) and the thiamine/TPP ratio were significantly reduced (Fig. 1G and H), while the intracellular thiamine level did not show significant changes (Fig. 1E). This suggests that the impact of CQ on intracellular thiamine homeostasis may be primarily mediated through its influence on TPP. Subsequently, we sought to investigate the effects of thiamine and TPP supplementation in combination with CQ treatment, with the aim of determining whether the toxicity associated with CQ was influenced by thiamine or TPP deficiency, as assessed by cell proliferation. As illustrated in Fig. 1I and J, concurrent administration of thiamine or TPP during CQ exposure markedly elevated the intracellular concentrations of both thiamine and TPP. Furthermore, the findings in Fig. 1K demonstrated that a 1 mM thiamine or TPP significantly mitigates CQ-induced toxicity, with TPP exhibiting greater efficacy than thiamine. Combining the effects of CQ on total thiamine and thiamine/TPP, these results suggest that CQ's toxic effects may be linked to a deficiency in TPP.

### 3.2 The effect of CQ on the metabolism and transport pathway of thiamine

**Fig. 2:**
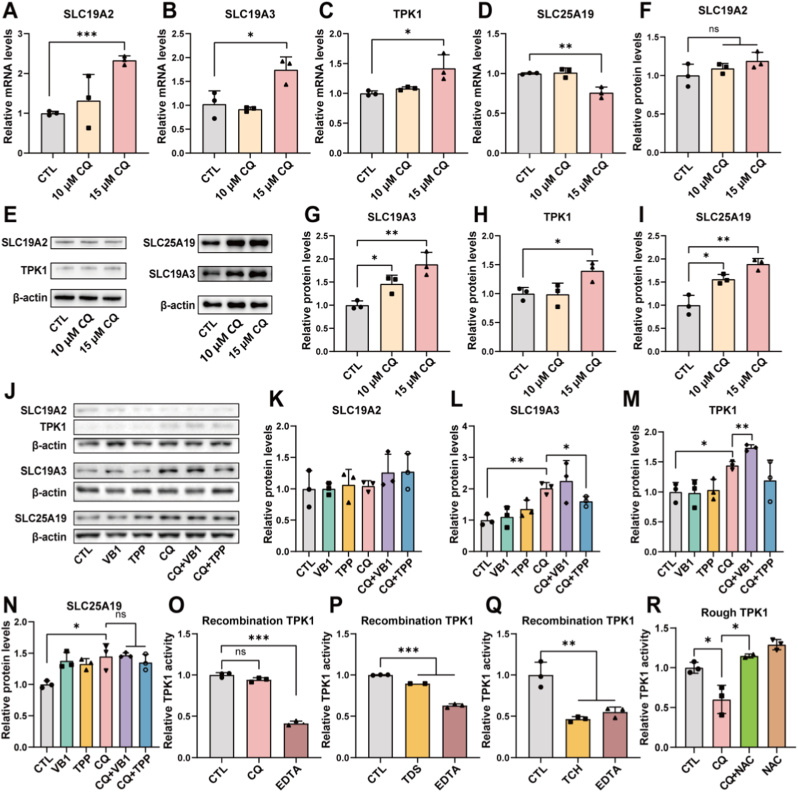
The effect of CQ on thiamine absorption and metabolic pathways in SH-SY5Y cells. (A-D) The impact of CQ treatment for 24 h on the mRNA levels of thiamine absorption and metabolism-related genes (*SLC19A2*, *SLC19A3*, *SLC25A19,* and *TPK1*) in SH-SY5Y cells. (E) The effect of 24-h CQ treatment on the protein levels of genes related to thiamine absorption and metabolism (SLC19A2, SLC19A3, SLC25A19, and TPK1) in SH-SY5Y cells. (F) Grayscale analysis bar chart of SLC19A2. (G) Grayscale analysis bar chart of SLC19A3. (H) Grayscale analysis bar chart of TPK1. (I) Grayscale analysis bar chart of SLC25A19. (J) The protein levels of genes related to thiamine absorption and metabolism (SLC19A2, SLC19A3, SLC25A19, and TPK1) in the groups of CTL, VB_1_, TPP, CQ, CQ + VB_1_, and CQ + TPP, where the concentration of CQ is 15 μM, and the concentrations of VB_1_ and TPP are both 1 mM. (K) Grayscale analysis bar chart of SLC19A2. (L) Grayscale analysis bar chart of SLC19A3. (M) Grayscale analysis bar chart of TPK1. (N) Grayscale analysis bar chart of SLC25A19. (F–I and K–N) β-actin was used as the internal reference protein. (O-Q) The effect of 15 μM CQ (O), 1 mM TDS (P), and 1 mM TCH (Q) on recombination TPK1 enzyme activity. (R) The relative TPK1 enzyme activity of SH-SY5Y cells in the CTL, CQ, CQ + NAC, and NAC groups. Data (n = 3) were presented as mean ± SE. ∗, P < 0.05; ∗∗, P < 0.01; ∗∗∗, P < 0.001.

Since CQ influenced the intercellular concentration of thiamine and TPP, we analyzed whether CQ affected the expression of genes associated with thiamine transport and metabolism. It is important to note that human cells cannot synthesize thiamine autonomously and must acquire it from their external environment. Thiamine is transported into the cell from the extracellular milieu via the transporters THTR1 (encoded by *SLC19A2*) and THTR2 (encoded by *SLC19A3*), and is subsequently phosphorylated by thiamine pyrophosphokinase 1 (*TPK1*) to form TPP within the cellular context [[24], [25], [26]]. TPP can then enter the mitochondria by the transport protein SLC25A19 to function as a coenzyme [27]. Our findings revealed that 15 μM CQ significantly increased the transcription levels of *SLC19A2,SLC19A3,* and *TPK1* (Fig. 2A–C), while inhibiting the transcription level of *SLC25A19* (Fig. 2D). Subsequently, we evaluated the protein expression levels of the affected genes and discovered that treatment with 15 μM CQ significantly elevated the protein level of SLC19A3, SLC25A19 and TPK1 (Fig. 2E–I), but had no significant effect on the protein expression level of SLC19A2 (Fig. 2F). However, these findings could not elucidate the above observed inhibitory effect of CQ on TPP and its implications for thiamine levels.

The effects of CQ on the expression of these genes and proteins may result from TPP deficiency. Therefore, we attempted to supplement thiamine or TPP during CQ treatment to determine the rationale for changes in the expression levels of thiamine transport and TPP synthesis genes. Supplementation with thiamine or TPP had no significant effect on SLC19A2 protein levels (Fig. 2K). Our findings indicated that TPP effectively mitigated the CQ-induced elevation in SLC19A3 and TPK1 protein levels (Fig. 2L). During CQ treatment, supplementation with thiamine further increased TPK1 expression (Fig. 2M). The CQ + TPP group showed a downward trend in TPK1 levels compared to the CQ group, but this was not significant (Fig. 2M). Supplementation with thiamine or TPP during CQ treatment did not significantly affect SLC25A19. Based on the previous HPLC data, it has been established that a concentration of 15 μM CQ increases intracellular thiamine levels (Fig. 1E). These data may suggest that the increased expression of SLC19A3 during CQ treatment is related to intracellular TPP deficiency, while the increased expression of TPK1 during CQ treatment may be associated with both intracellular thiamine accumulation and TPP deficiency. The modulation of these genes by CQ may represent a feedback regulatory mechanism resulting from alterations in TPP and thiamine concentrations, suggesting that CQ does not directly affect intracellular TPP and thiamine levels through inhibiting expression levels of the thiamine transport and TPP synthesis pathways.

We conducted additional investigations into the impact of CQ on the functional activity of these pathways. Observing an elevation in thiamine concentrations during CQ administration (Fig. 1E), we hypothesize that CQ predominantly inhibits TPK1 enzymatic activity rather than impeding cellular thiamine uptake. Consequently, we evaluated the effect of CQ on TPK1 enzymatic activity. However, the data, as illustrated in Fig. 2O, demonstrate that CQ does not inhibit the activity of recombination TPK1. Notably, we observed that oxidative byproducts of thiamine, specifically thiamine disulfide and thiochrome, can inhibit TPK1 activity (Fig. 2P and Q), suggesting that CQ may influence TPP levels by inducing oxidative stress.

To better evaluate the impact of CQ-induced oxidative stress on intracellular TPK1 enzyme activity, we administered the antioxidant N-acetylcysteine (NAC) during CQ treatment. Subsequently, cellular extracts were prepared to assess the crude enzymatic activity of TPK1. The results demonstrated that CQ significantly inhibited TPK1 activity, whereas NAC supplementation restored the enzyme's function (Fig. 2R). These findings suggest that CQ does not directly inhibit TPK1 activity; rather, it may induce the production of TPK1 inhibitors through oxidative stress mechanisms. Recent studies have identified UTP as a critical substrate for TPK1, and UTP levels influence TPP synthesis [28]. We analyzed UTP concentrations using liquid chromatography-mass spectrometry and found that CQ does not alter UTP levels (Fig. S2).

### 3.3 CQ induces oxidative stress and facilitates the oxidation of TPP

**Fig. 3:**
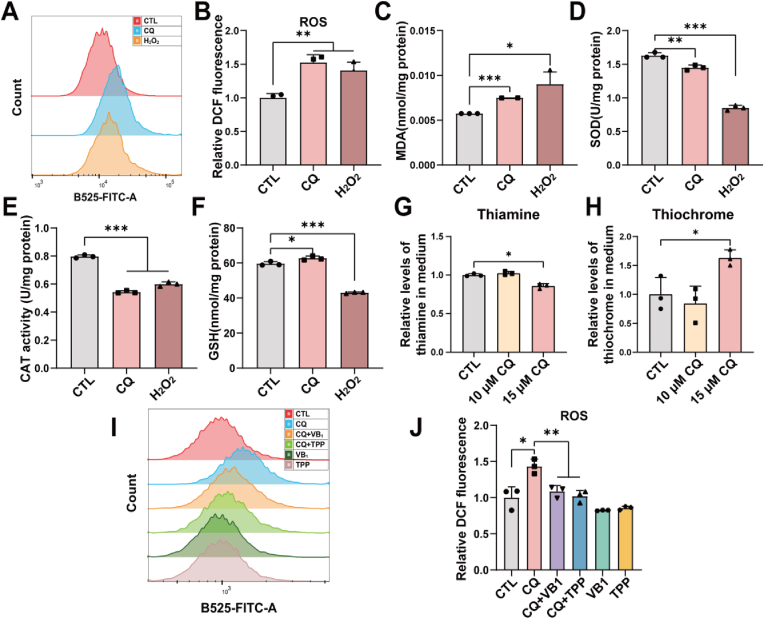
CQ induces oxidative stress and facilitates the oxidation of TPP. (A-F) CQ treatment for 24 h increased levels of ROS (A and B), MDA (C), and total GSH (F), while decreasing the activity of SOD (D) and CAT (E) in SH-SY5Y cells. (A-F) The concentration of CQ was 15 μM, with 600 μM H_2_O_2_ as the positive control. (G and H) The effect of CQ (10, 15 μM) treatment for 24 h on total thiamine (G) and thiochrome (H) levels in SH-SY5Y cell culture medium. (I and J) The level of ROS in the groups of CTL, CQ, CQ + VB_1_, CQ + TPP, VB_1_, and TPP, where the concentration of CQ is 15 μM, and the concentrations of VB_1_ and TPP are both 1 mM. (B and J) The Y-axis of the bar graph represents the mean fluorescence intensity of each group of DCF relative to the control group. Data (n = 3) were presented as mean ± SE. ∗, P < 0.05; ∗∗, P < 0.01; ∗∗∗, P < 0.001.

Previous studies have shown that CQ induces ROS production, leading to oxidative stress [29]. The preceding data also suggest that CQ may induce oxidative stress, leading to the generation of thiamine oxidation products that inhibit TPK1 enzyme activity (Fig. 2P and Q). Herein, we measured common oxidative stress indicators, including ROS levels, malondialdehyde (MDA) levels, superoxide dismutase (SOD) activity, catalase (CAT) activity, and total glutathione (GSH) levels. From our data, CQ significantly elevated intracellular levels of ROS and MDA, while concurrently reducing the enzymatic activities of SOD and CAT (Fig. 3A–E). However, it is noteworthy that CQ actually increased total GSH levels (Fig. 3F). Our prior research demonstrated that low concentrations of CQ can mitigate oxidative stress in yeast by enhancing total intracellular GSH levels [30]. The findings presented in this study indicate that elevated concentrations of CQ also promote an increase in GSH levels during oxidative stress induction, suggesting that this effect may be an intrinsic property of CQ. The results of ROS, MDA, SOD, and CAT indicate that CQ elevates intracellular oxidative levels, thereby inducing oxidative stress. It was reported that thiamine can act as a reducing agent to eliminate ROS [31,32] and can be oxidized by oxidants into thiochrome and thiamine disulfide under different conditions [33]. We speculated that TPP may be degraded by ROS generated by CQ treatment. We measured thiamine oxidation product, thiochrome, in the culture medium using thiochrome fluorescence detection. It was found that CQ increased thiochrome levels in the culture medium (P = 0.0276) while decreasing the total thiamine level in the medium (P = 0.0025) (Fig. 3G and H). This result indicates that CQ treatment increased thiamine consumption by cells and enhanced the production of thiamine oxidation products. The above results are consistent with our hypothesis that CQ induces oxidative stress, increases intracellular oxidative levels, and leads to the oxidation of thiamine or TPP. Furthermore, it is essential to highlight that our prior findings indicated that the oxidative derivatives of thiamine, specifically disulfide thiamine and thiochrome, possess the capability to inhibit the enzymatic activity of TPK1 (Fig. 2P and Q). Consequently, an elevation in cellular oxidative products is likely to further intensify the deficiency of TPP. Subsequently, we evaluated the ROS-scavenging properties of thiamine and TPP. The findings demonstrated that both thiamine and TPP markedly decreased the ROS levels generated by CQ treatment (Fig. 3I and J).

### 3.4 Antioxidants restore TPP levels by inhibiting oxidative stress

**Fig. 4:**
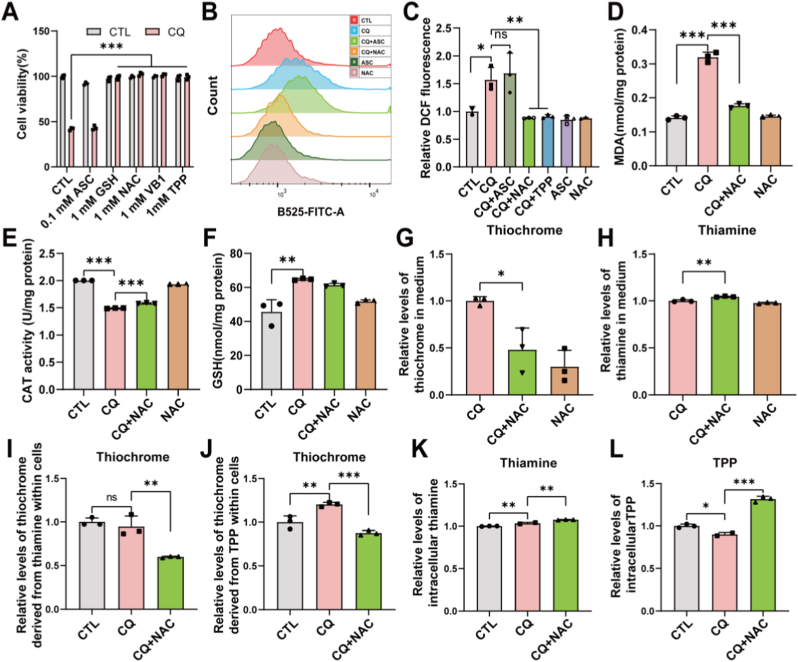
High concentrations of thiamine or TPP inhibit oxidative stress caused by CQ in SH-SY5Y cells. (A) The bar graph shows the effects of 0.1 mM ASC, 1 mM GSH, 1 mM NAC, 1 mM VB1, and 1 mM TPP on the proliferation activity of SH-SY5Y cells in the presence or absence of CQ. (B and C) The relative ROS levels in the groups of the CTL, CQ, CQ + ASC, CQ + NAC, CQ + TPP, ASC, and NAC. (D –F) The bar graph represents the cellular MDA levels (D), CAT enzyme activity (E), and total GSH levels (F) of SH-SY5Y cells in the CTL, CQ, CQ + NAC, and NAC groups. (G) The bar chart shows the relative levels of thiochrome in the culture medium of the CQ, CQ + NAC, and NAC groups. (H) The bar chart shows the relative levels of thiamine in the culture medium of the CQ, CQ + NAC, and NAC groups. (I) The bar graph represents the relative levels of intracellular thiochrome derived from thiamine of SH-SY5Y in the CTL, CQ, and CQ + NAC groups. (J) The bar graph represents the relative levels of intracellular thiochrome derived from TPP of SH-SY5Y in the CTL, CQ, and CQ + NAC groups. (K) The bar graph represents the relative levels of intracellular thiamine in the CTL, CQ, and CQ + NAC groups. (L) The bar graph represents the relative levels of intracellular TPP in the CTL, CQ, and CQ + NAC groups. (D-L) The concentration of CQ is 15 μM. The concentration of NAC is 1 mM. Data (n = 3) were presented as mean ± SE. ∗, P < 0.05; ∗∗, P < 0.01; ∗∗∗, P < 0.001.

Based on our hypothesis that CQ induces TPP deficiency through the promotion of oxidative stress, we posited that the application of alternative antioxidants to mitigate cellular oxidative stress would restore TPP levels. Initially, we evaluated the inhibitory effects of various antioxidants on CQ-induced toxicity. The results obtained from the CCK8 assay indicated that GSH and NAC, similar to thiamine and TPP, effectively attenuated CQ toxicity, whereas ascorbic acid (ASC) did not exhibit such protective effects (Fig. 4A). It is noteworthy that the supplementation of GSH can mitigate the cytotoxic effects of CQ (Fig. 4A). However, it has been previously established that CQ can also elevate GSH levels (Fig. 3F). This suggests that the inhibition of CQ-induced toxicity is contingent upon specific GSH concentration thresholds. While CQ is capable of enhancing GSH levels, the extent of this increase may be inadequate to counteract the cytotoxicity associated with elevated concentrations of CQ. Consequently, NAC was selected as the antioxidant of interest for further investigation, with ASC and TPP serving as control comparators to examine NAC's impact on CQ-induced ROS generation. Flow cytometric analysis revealed that NAC, akin to TPP, significantly diminished the CQ-induced elevation of ROS levels, while ASC showed no significant influence (Fig. 4B and C). We conducted further investigations into the restorative effects of NAC on oxidative stress-related biomarkers induced by CQ. The findings indicated that NAC significantly mitigated the elevation of MDA levels associated with CQ treatment (Fig. 4D). NAC also restored CAT enzyme activity (Fig. 4E). Additionally, there was no notable alteration in GSH levels when CQ was co-administered with NAC compared to CQ treatment alone (Fig. 4F). Taken together, our study identifies NAC as an effective antioxidant capable of counteracting CQ-induced toxicity and inhibiting oxidative stress.

We then further examined the recovery of TPP levels by NAC under the CQ treatment. We treated cells with both CQ and NAC, then measured total thiamine and TPP levels in the culture medium. The results demonstrated that co-treatment increased thiamine levels (P = 0.0044) while concurrently decreasing thiochrome levels in the culture medium (P = 0.0190) (Fig. 4G and H). This suggests that NAC may reduce thiamine oxidation or TPP oxidation during CQ exposure, thereby reducing thiamine consumption by cells. Further analysis by HPLC to detect intracellular oxidation products revealed that CQ significantly elevated TPP oxidation products, but did not affect thiamine oxidation products (Fig. 4I and J). NAC supplementation effectively reversed the increase in TPP oxidation products induced by CQ and also diminished the levels of thiamine oxidation products (Fig. 4I and J). It is important to note that, while HPLC detection of intracellular oxidation products of thiamine and its derivatives has demonstrated that CQ elevates the oxidative products of TPP, our analysis was limited to measuring thiochrome levels in the culture medium. This limitation arises from the inherent instability of TPP oxidative products, which are readily hydrolyzed by phosphatases commonly found in the extracellular environment, resulting in their conversion to thiochrome [34].

Subsequently, we assessed the recovery of TPP levels during CQ treatment with NAC supplementation. The results revealed that the addition of NAC during CQ treatment significantly elevated both TPP and thiamine levels (Fig. 4K and L). The results presented above indicate that CQ induces TPP oxidation via oxidative stress mechanisms, ultimately leading to a depletion of TPP levels.

### 3.5 CQ induces mitochondrial dysfunction and metabolic reprogramming

**Fig. 5:**
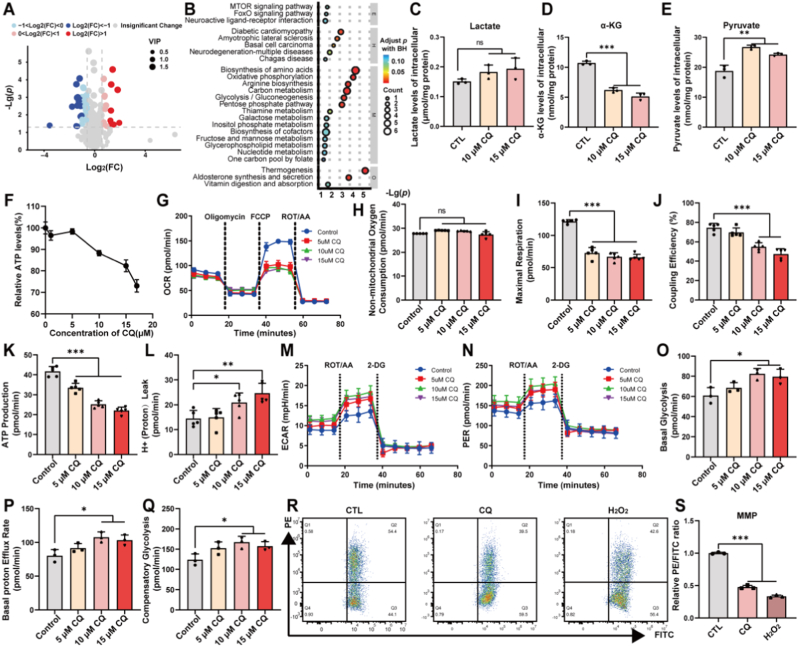
CQ induces mitochondrial dysfunction and metabolic reprogramming. (A) The volcano plot shows the differential metabolites within cells after 15 μM CQ treatment of SH-SY5Y for 24 h. Different colors represent the metabolites' log2 fold change, and the size indicates the metabolites' VIP scores. (B) The bubble chart represents the KEGG functional enrichment of differential metabolites. (C) Intracellular lactate levels. (D) Intracellular α-ketoglutarate levels. (E) Intracellular pyruvic acid levels. (C-E) After treating SH-SY5Y cells with 10 μM and 15 μM CQ for 24 h, detection was performed using kits. (F) The line graph shows the effect of different concentrations of CQ treatment for 24 h on ATP levels in SH-SY5Y cells. (G) Using the mito stress assay kit to assess the effect of CQ on mitochondrial oxidative phosphorylation in SH-SY5Y cells. FCCP, carbonyl cyanide 4-(trifluoromethoxy) phenylhydrazone. ROT, rotenone. AA, antimycin. OCR, oxygen consumption rate. (H-L) Effects of different concentrations of CQ treatment on Non-Mitochondrial Oxygen Consumption (H), Maximal Respiration (I), Couple Efficiency (J), ATP Production (K), and Proton Leak (L) in SH-SY5Y Cells. (M and N) Using a glycolysis rate assay kit to detect the effects of different concentrations of CQ on the glycolysis levels of SH-SY5Y cells. ECAR, extracellular acidification rate (M). PER, proton efflux rate (N). (O-Q) Effects of different concentrations of CQ treatment on Basal Glycolysis (O), Basal Proton Efflux Rate (P), Compensatory Glycolysis (Q) in SH-SY5Y Cells. (R) Using flow cytometry with the JC-1 method to detect the effect of CQ on the mitochondrial membrane potential of SH-SY5Y cells. (S) The bar graph shows the effect of CQ on the mitochondrial membrane potential of SH-SY5Y cells. Data (n = 3) were presented as mean ± SE. ∗, P < 0.05; ∗∗, P < 0.01; ∗∗∗, P < 0.001.

To further investigate the effects of CQ on cellular functions, we conducted an in-depth analysis of high-throughput metabolomics data from CQ-treated SH-SY5Y cells. First, we analyzed differential metabolites between sample groups. As shown in the volcano plot, differential metabolites were selected using the criteria of fold change (FC) > 1.5 or FC < 0.667, and P-value <0.05 (Fig. 5A). In the plot, red dots represent upregulated metabolites, blue dots represent downregulated metabolites, and the size of each dot corresponds to the metabolite's VIP value (Fig. 5B). To further understand the metabolic pathways involved, we performed KEGG pathway enrichment analysis on the differential metabolites and found significant enrichment in energy metabolism-related pathways, including oxidative phosphorylation and glycolysis/gluconeogenesis. Additionally, thiamine-related pathways, such as the pentose phosphate pathway and thiamine metabolism, were significantly enriched (Fig. 5B). TPP is a coenzyme of pyruvate dehydrogenase and α-ketoglutarate dehydrogenase. A deficiency in TPP may lead to the accumulation of pyruvate, lactate, and α-ketoglutarate. We measured lactate, pyruvate, and α-ketoglutarate levels in SH-SY5Y cells treated with 15 μM CQ for 24 h. The results showed that CQ treatment did not have a significant effect on lactate levels, although there was a trend toward an increase (Fig. 5C). CQ treatment significantly increased pyruvate levels (Fig. 5D) but decreased α-ketoglutarate levels (Fig. 5E). The accumulation of pyruvate is consistent with TPP deficiency, while the reduction in α-ketoglutarate may be due to decreased downstream products resulting from reduced pyruvate entering the citric acid cycle. Herein, through metabolomics, we also discovered that CQ affects oxidative phosphorylation, glycolysis, and gluconeogenesis. Therefore, we further analyzed the impact of CQ on energy metabolism. Using an ATP assay kit to measure its effect on ATP levels in SH-SY5Y cells, the results showed that CQ at concentrations above 10 μM significantly reduced intracellular ATP levels (Fig. 5F).

To further evaluate the effects of CQ on cellular energy metabolism, we used the Cell Mito Stress Test Kit from Seahorse to assess mitochondrial energy metabolism (Fig. 5G). The results showed that treatment with 5-15 μM CQ did not significantly affect non-mitochondrial oxygen consumption in SH-SY5Y cells (Fig. 5H). However, it significantly reduced the mitochondrial maximal respiratory capacity (Fig. 5I), indicating mitochondrial dysfunction. Further analysis revealed that 10-15 μM CQ significantly decreased coupling efficiency of respiration and significantly increased proton leak rate, suggesting that CQ may disrupt mitochondrial membrane integrity (Fig. 5J–L). We conducted an additional assessment of mitochondrial membrane integrity using JC-1 dye. The data showed that 15 μM CQ, similar to the positive control H_2_O_2_, significantly reduced mitochondrial membrane potential and disrupted mitochondrial membrane integrity (Fig. 5R and S). The data of glycolytic rate indicated that 10-15 μM CQ significantly increased basal glycolysis levels and basal proton efflux rate (Fig. 5M–O), suggesting that CQ elevated cellular glycolytic activity. Additionally, CQ increased compensatory glycolysis levels (Fig. 5Q), indicating that CQ treatment enhanced glycolysis in cells with impaired mitochondrial function. These results collectively demonstrate that CQ inhibits mitochondrial oxidative phosphorylation function and causes mitochondrial damage, leading to a compensatory increase in glycolytic function.

### 3.6 TPP restores mitochondrial damage caused by CQ

**Fig. 6:**
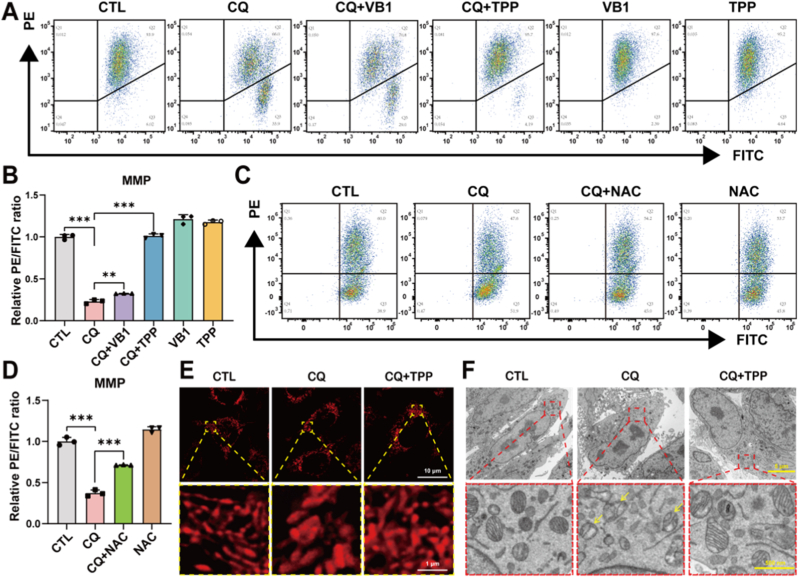
TPP restored mitochondrial damage caused by CQ in SH-SY5Y. (A) Using flow cytometry with the JC-1 method to detect the mitochondrial membrane potential of SH-SY5Y cells in the CTL, CQ, CQ + VB1, CQ + TPP, VB1, and TPP groups. (B) Bar graph showing the mitochondrial membrane potential of SH-SY5Y cells in the CTL, CQ, CQ + VB1, CQ + TPP, VB1, and TPP groups. (C) Using flow cytometry with the JC-1 method to detect the mitochondrial membrane potential of SH-SY5Y cells in the CTL, CQ, CQ + NAC, and NAC groups. (D) Bar graph showing the mitochondrial membrane potential of SH-SY5Y cells in the CTL, CQ, CQ + NAC, and NAC groups. (E) Using laser confocal microscopy to image mitochondria in SH-SY5Y cells labeled with MitoTracker Red. (F) Using transmission electron microscopy to capture the mitochondrial morphology of SH-SY5Y cells in the CTL, CQ + TPP, and TPP groups. Data (n = 3) were presented as mean ± SE. ∗, P < 0.05; ∗∗, P < 0.01; ∗∗∗, P < 0.001.

Earlier studies showed that CQ-induced metabolic reprogramming is associated with TPP deficiency. We hypothesized that CQ-induced mitochondrial damage is also related to TPP. The preceding results indicated that CQ disrupts cellular energy metabolism and damages mitochondrial structure. We hypothesized that these effects may be associated with the deficiency of TPP. To investigate this, we examined whether TPP supplementation could restore mitochondrial integrity compromised by CQ exposure. Using the JC-1 dye, we examined the restorative effects of thiamine and TPP on the mitochondrial membrane potential decline caused by CQ. The results showed that both thiamine and TPP significantly restored the CQ-induced decrease in mitochondrial membrane potential, with TPP demonstrating a notably stronger effect than thiamine (Fig. 6A and B). We stained mitochondria with Mitotracker and captured images using laser confocal microscopy, observing that CQ altered mitochondrial morphology, changing it from a normal elongated shape to a short and thick form, while TPP was able to partially restore the mitochondrial morphology (Fig. 6E). To further characterize CQ-induced mitochondrial damage and the protective effect of TPP, we used transmission electron microscopy to examine mitochondrial ultrastructure. The results showed that after CQ treatment, mitochondrial cristae disappeared (yellow arrows), indicating severe mitochondrial damage, whereas TPP reduced this damage and helped mitochondria return toward normal morphology (Fig. 6F). In our previous research, we found that NAC can restore TPP levels; therefore, we speculated that NAC might have a protective effect similar to TPP. The results confirmed that NAC also restored the CQ-induced decline in mitochondrial membrane potential (Fig. 6C and D). Taken together, our data indicate that CQ-induced mitochondrial damage is associated with TPP deficiency.

### 3.7 TPP reverses the metabolic reprogramming induced by CQ

**Fig. 7:**
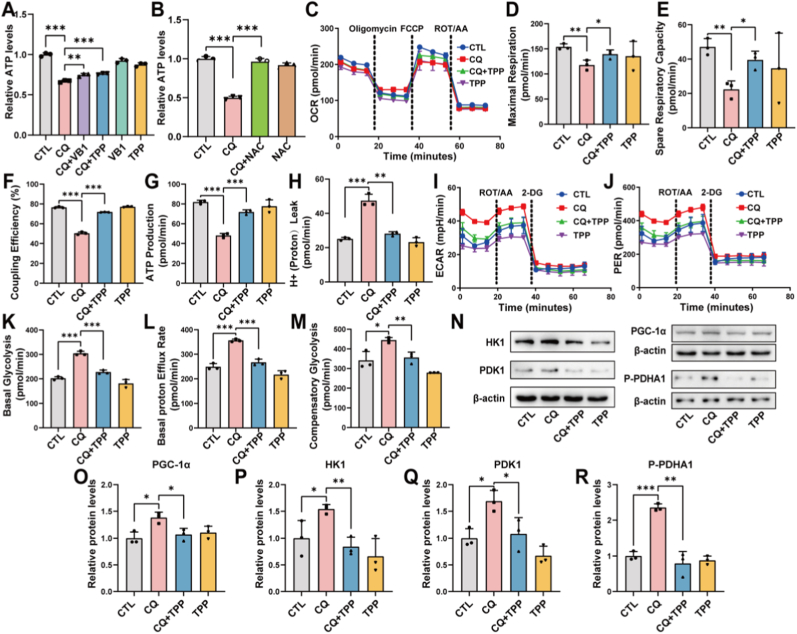
TPP reversed the metabolic reprogramming induced by CQ. (A) The bar graph represents the ATP levels of SH-SY5Y cells in the CTL, CQ, CQ + VB1, CQ + TPP, VB1, and TPP groups. (B) The bar graph represents ATP levels in SH-SY5Y cells of the CTL, CQ, CQ + NAC, and NAC groups. (C) The effect of CQ on mitochondrial oxidative phosphorylation in the CTL, CQ, CQ + TPP, and TPP groups was assessed by the mito stress assay kit. (D-H) Effects of Different Concentrations of CQ Treatment on Maximal Respiration (D), Spare Respiratory Capacity (E), Couple Efficiency (F), ATP Production (G), and Proton Leak (H) in the CTL, CQ, CQ + TPP, and TPP groups. (I and J) The glycolysis levels in SH-SY5Y cells of the CTL, CQ, CQ + TPP, and TPP groups were detected by the glycolysis rate assay kit. ECAR, extracellular acidification rate (I). PER, proton efflux rate (J). (K-M) Effects of different concentrations of CQ treatment on Basal Glycolysis (K), Basal Proton Efflux Rate (L), Compensatory Glycolysis (M) in SH-SY5Y Cells of the CTL, CQ, CQ + TPP, and TPP groups. (N) Western Blotting detection of HK1, PGC-1α, PDK1, and p-PDHA1 protein levels in SH-SY5Y cells of the CTL and CQ, CQ + TPP, and TPP groups. (O-Q) The bar graph represents the relative levels of PGC-1α (O), HK1 (P), PDK1 (Q), and P-PDHA1 (R) proteins in the CTL, CQ, CQ + TPP, and TPP groups. The bar graph represents the relative levels of p-PDHA1. Data (n = 3) were presented as mean ± SE. ∗, P < 0.05; ∗∗, P < 0.01; ∗∗∗, P < 0.001.

Our findings above indicate that CQ induces a deficiency of TPP and alters energy metabolism pathways. Given that TPP is a crucial coenzyme in the citric acid cycle, we hypothesized that CQ-induced disruption of energy metabolism may be associated with TPP deficiency. We evaluated the restorative effects of TPP supplementation on CQ-induced metabolic disturbances. Using an ATP assay kit, we measured intracellular ATP levels in SH-SY5Y cells. The results demonstrated a significant reduction in ATP levels following CQ treatment, whereas supplementation with thiamine, TPP, or NAC markedly restored intracellular ATP concentrations (Fig. 7A and B), suggesting that both compounds mitigate the impact of CQ on cellular energy metabolism. Further assessment of oxidative phosphorylation via Seahorse analysis revealed that CQ treatment significantly decreased maximal respiration (Fig. 7D), spare respiratory capacity (Fig. 7E), coupling efficiency (Fig. 7F), and ATP production (Fig. 7G). Notably, TPP supplementation substantially reversed these declines (Fig. 7C–G). Additionally, CQ exposure led to a significant increase in proton leak, which was effectively reduced by TPP supplementation (Fig. 7H). These findings indicate that TPP replenishment can restore CQ-impaired oxidative phosphorylation and preserve mitochondrial membrane integrity.

Previously, we observed a compensatory upregulation of glycolysis following CQ treatment (Fig. 5M–Q). Given the current evidence that TPP restores oxidative phosphorylation, we posited that TPP might also normalize the CQ-induced enhancement of glycolytic activity. Seahorse analysis of glycolytic rate confirmed that TPP supplementation attenuated the CQ-induced increase in glycolysis (Fig. 7I–M). Moreover, we examined the effects of TPP supplementation on key proteins involved in energy metabolism pathways. Hexokinase is the first rate-limiting enzyme in glycolysis [35]. PGC-1α is an important transcriptional coactivator in gluconeogenesis [36]. PDK1 can inhibit the citric acid cycle by phosphorylating the pyruvate dehydrogenase subunits PDHA1 and PDHA2 [37]. CQ treatment elevated the expression levels of PGC-1α, HK1, PDK1, and phosphorylated PDHA1 (Fig. 7N–R). Importantly, TPP supplementation effectively normalized the expression of these proteins. Collectively (Fig. 7N–R), these results suggest that TPP supplementation ameliorates CQ-induced disruptions in cellular energy metabolism by restoring mitochondrial function and modulating metabolic regulatory proteins. These findings indicate that TPP supplementation can reverse the metabolic reprogramming induced by CQ. In conjunction with the earlier observation that CQ causes TPP deficiency, these results suggest that CQ may induce metabolic reprogramming by depleting TPP.

### 3.8 CQ induces TPP deficiency in the brain of AD mice via oxidative stress

**Fig. 8:**
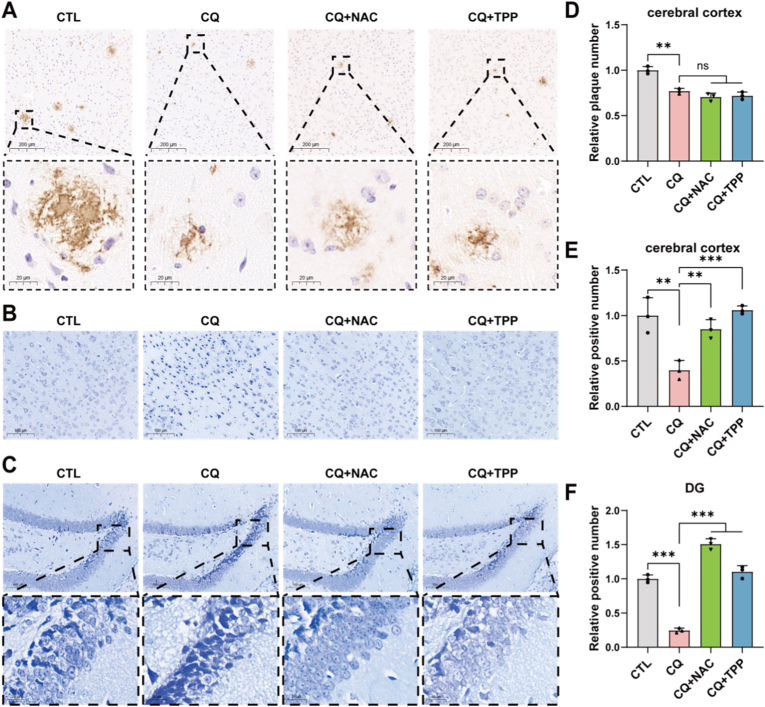
NAC/TPP mitigates neurodegeneration induced by CQ in AD mice. (A) Immunohistochemical assessment of Aβ42 in paraffin sections of mouse brain tissue to evaluate amyloid plaques in an AD model; (B, C) Evaluation of neuronal damage in the cerebral cortex (B) and dentate gyrus of the hippocampus DG (C) using Nissl staining on paraffin sections of mouse brain tissue. (D) The bar chart shows the relative plaque number of AD model mice cerebral cortex in CTL and CQ, CQ + NAC, and CQ + TPP groups. (E-F) The bar chart shows the relative Nissl staining positive cell number of AD model mice cerebral cortex (E) and hippocampus DG (F) in CTL and CQ, CQ + NAC, and CQ + TPP groups. Data were presented as mean ± SE. ∗∗, P < 0.01; ∗∗∗, P < 0.001.

To further investigate the neurotoxicity mechanism of CQ under physiological conditions, particularly in the context of AD treatment, we administered CQ to a mouse model of AD (APP/PS1). We used a higher dose of 90 mg/kg/day to simulate the potential neurotoxic effects of CQ. The immunohistochemical analysis of Aβ42, as illustrated in Fig. 8A and D, showed that the CQ-treated group exhibited a reduction in both the number and size of amyloid plaques compared to the control group without CQ treatment in the cerebral cortex. This finding suggests that this CQ concentration still exerts amyloid plaque-clearing activity, consistent with previous literature [1,2]. However, Nissl staining of cerebral cortex and hippocampus dentate gyrus (DG) showed that the CQ-treated group displayed more pronounced cell shrinkage and nuclear pyknosis than the control group, suggesting that this CQ concentration exacerbated neuronal damage in the AD model mice cerebral cortex (Fig. 8B and E) and DG (Fig. 8C and F). CQ has similar effects in the hippocampal Ammon's horn areas (CA) 1 and 3 as in the cerebral cortex and DG regions, although statistical analysis shows that CQ does not significantly reduce the number of positive cells in CA1 and CA3 (Fig. S3). In addition, we used Fluoro-Jade C staining to assess the extent of neurodegeneration, and the results showed that CQ did not exacerbate neuronal degeneration, which may be related to its ability to clear amyloid proteins (Fig. S11).

The detection of thiamine transport and TPP synthesis gene expression levels in the brain tissue of AD model mice treated with CQ (Fig. S2) showed that oral administration of CQ significantly increased the mRNA levels of *SLC19A3* and *SLC25A19* in mouse brain tissue, and did not inhibit the mRNA levels of *SLC19A2* and *TPK1*; supplementation with TPP/NAC during oral CQ administration restored the level of *SLC19A3* (Fig. S4). These results are consistent with the in vitro findings (Fig. 2A–D), indicating that CQ does not induce TPP deficiency by suppressing the expression of these genes.

**Fig. 9:**
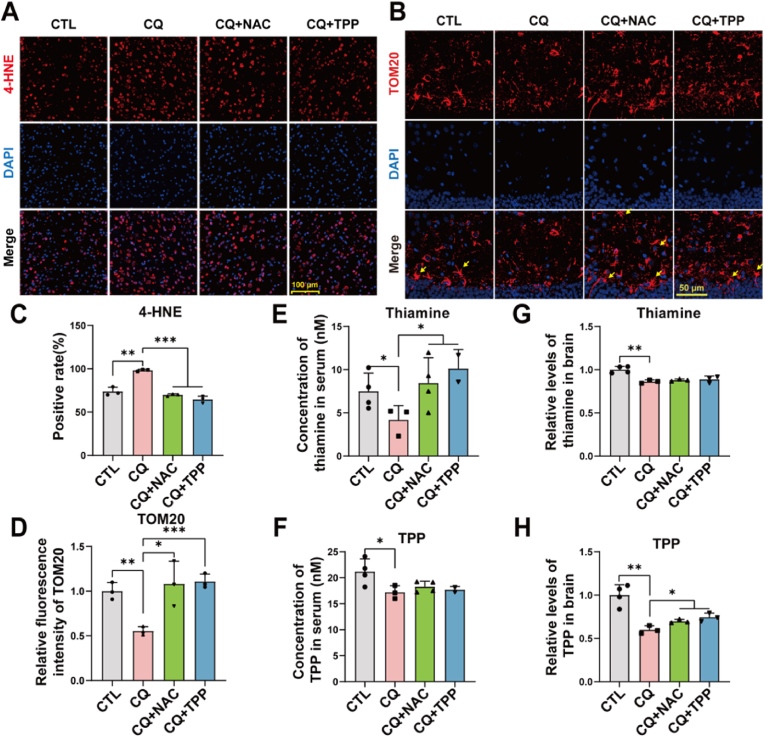
CQ induces TPP deficiency in the brain of AD mice via oxidative stress. (A) The oxidative stress levels in the cerebral cortex of AD model mice under different drug treatments were assessed using 4-HNE immunofluorescence. (B) The TOM20 levels in the hippocampus of AD model mice under different drug treatments were assessed using immunofluorescence. (C) The bar chart shows the positive rate in CTL and CQ, CQ + NAC, and CQ + TPP groups. The positivity rate was calculated by the ratio of 4-HNE-labeled cells to DAPI-labeled cells in three randomly selected fields. (D) The bar chart shows the relative fluorescence intensity of TOM20 in CTL and CQ, CQ + NAC, and CQ + TPP groups. (E-F) The bar graph shows the levels of thiamine (E) and TPP (F) in the serum from the CTL, CQ, CQ + NAC, and CQ + TPP groups. (G-H) The bar graph shows the levels of thiamine (G) and TPP (H) in the brain from the CTL, CQ, CQ + NAC, and CQ + TPP groups. Thiamine and TPP were extracted from intact hemispheres. Data were presented as mean ± SE. ∗, P < 0.05; ∗∗, P < 0.01; ∗∗∗, P < 0.001.

4-Hydroxynonenal (4-HNE) is a deleterious byproduct of fatty acid peroxidation [38]. Exposure of cells to oxidative stress or damage triggers lipid peroxidation,leading to the formation of 4-HNE from fatty acids [38]. Immunofluorescence detection of 4-HNE levels revealed an increase in the proportion of cells exhibiting oxidative stress following CQ administration, indicating that CQ enhances oxidative stress in the cerebral cortex (Fig. 9A and C). The induction effect of CQ on oxidative stress in the CA1, CA3, and DG regions is similar to that in the cerebral cortex (Fig. S5). To assess mitochondrial damage in brain tissue cells, we employed immunofluorescence to detect TOM20 expression in DG (Fig. 9B and D), CA1, CA3, and the cerebral cortex (Fig. S6). TOM20 is a structural marker of the mitochondrial outer membrane and can be used to determine the spatial distribution of mitochondria within tissues or cells [39,40]. Additionally, variations in the intensity and morphological characteristics of TOM20 signals can be used to assess morphological alterations associated with mitochondrial damage [39,40]. Immunofluorescence showed that the TOM20 signal filled the cytoplasm and outlined the contours of the neurons from the different regions in the control group. While the TOM20 signal was more fragmented after the treatment of CQ, suggesting that the number and distribution of mitochondria within the cells were disturbed (Fig. 9B, yellow arrows, Fig. S6). Additionally, we examined the transcriptional levels of *Drp1* and *Mfn2* in mice brain tissue (Fig. S7). The data indicate that following CQ treatment, the expression levels of these two genes were significantly upregulated. The upregulation of Drp1 is generally associated with mitochondrial fragmentation, whereas the upregulation of Mfn2 is typically linked to mitochondrial remodeling in response to stress [[41], [42], [43]]. To further investigate the impact of CQ on thiamine levels in mice, we quantified thiamine and TPP concentrations in both serum and brain tissue. The data demonstrated significant decreases in thiamine and TPP levels in both serum and brain following CQ treatment, suggesting that CQ induces TPP deficiency in the mouse serum and brain (Fig. 9E–H). In conclusion, our study provides further evidence that CQ induces neurotoxicity in an AD mouse model, characterized by increased oxidative stress and TPP deficiency.

### 3.9 NAC/TPP mitigates neurodegeneration induced by CQ in AD mice

To further investigate the relationship among CQ-induced oxidative stress, TPP deficiency, and nerve damage, and to explore treatment regimens that do not compromise CQ's ability to clear amyloid plaques while effectively reducing CQ-induced neurotoxicity, we conducted experiments in which subjects were supplemented with NAC or TPP during CQ administration. Firstly, to evaluate whether combined medication would impair CQ's ability to clear amyloid plaques, we conducted an Aβ42 immunohistochemistry experiment. The results showed that the level of amyloid plaques in the cerebral cortex of mice treated with CQ combined with NAC/TPP was similar to that in the CQ group (Fig. 8A and D), indicating that the combined treatment did not diminish CQ's amyloid plaque-clearing ability. Nissl staining revealed that both NAC and TPP supplementation mitigated CQ-induced nerve damage in the cerebral cortex (Fig. 8B and E), DG (Fig. 8C and F), CA1 and CA3 (Fig. S3), suggesting a link between CQ-induced nerve damage, oxidative stress, and TPP deficiency. Subsequently, we assessed oxidative stress and TPP levels in mice receiving NAC or TPP supplementation. Immunofluorescence analysis for 4-HNE showed that the administration of NAC or TPP, alongside CQ, significantly reduced oxidative stress in the mice's cerebral cortex (Fig. 9A and C) and hippocampus (Fig. S5), consistent with previous in vitro results. TOM20 immunofluorescence showed that the combination of NAC or TPP with CQ could partially reverse the morphological alterations of damaged mitochrondria in DG (Fig. 9B) and cerebral cortex (Fig. S6). These findings indicate that CQ treatment leads to a reduction in neuronal cells and mitochondrial damage in the mouse brain, while both NAC and TPP mitigate the damage induced by CQ, with NAC demonstrating a more pronounced protective effect than TPP. Serum analyses indicated that both NAC and TPP supplementation effectively restored serum thiamine levels, although TPP levels were not fully restored (Fig. 9E and F). The possible explanation is that CQ induced TPP deficiency by oxidizing TPP, thereby enhancing thiamine uptake and consumption in SH-SY5Y cells (Fig. 3G and H). In the animal studies, the observed decrease in serum thiamine levels in the CQ group aligned with the previously noted increase in thiamine consumption (Fig. 9E). The supplementation of NAC or TPP fundamentally diminished the oxidation of TPP within the tissues, thereby reducing the serum-derived thiamine uptake and ultimately leading to the restoration of serum thiamine levels (Fig. 9E). After administration by gavage, intestinal phosphatases hydrolyze TPP into thiamine, which is then absorbed through the mucosal membrane [44], thereby rendering supplementation with NAC or TPP ineffective in elevating TPP levels [[45], [46], [47], [48]]. Given that TPP is metabolized and oxidized within tissues, we investigated the impact of NAC and TPP supplementation on thiamine and TPP concentrations in brain tissues. The findings revealed that both NAC and TPP supplementation significantly mitigated TPP deficiency induced by CQ in brain tissues (Fig. 9H). Based on changes in thiamine and TPP levels in serum after NAC or TPP supplementation, we conclude that such supplementation increases serum thiamine, enhances its supply to the brain, and leads to higher TPP production in brain tissue. Corroborating the results of 4-HNE analysis in brain tissue, these data suggest that inhibition of oxidative processes triggered by CQ can restore TPP levels in brain tissues. In addition, we examined oxidative stress status and TPP levels in brain tissues of wild-type mice, and the results showed that CQ did not induce TPP deficiency (Figs. S8–9) or oxidative stress (Fig. S10) in the brain tissues of wild-type mice. This indicated that CQ-induced TPP deficiency in animals is also associated with susceptibility to oxidative stress. In conclusion, our study further substantiates that CQ induces TPP deficiency via oxidative stress in AD model mice, ultimately leading to neuronal damage.

## 4 Discussion

CQ is a quinoline-based compound with potential therapeutic applications in neurodegenerative diseases. However, its clinical use is limited due to its associated neurotoxicity. Previous studies have suggested that this neurotoxicity may be linked to decreased levels of active vitamin B12 and to inhibition of proteasome activity [[49], [50], [51]]. In our study, we reported, for the first time, that CQ disrupts intracellular TPP levels, establishing a link between CQ-induced neurotoxicity and TPP deficiency. In the context of thiamine deficiency, factors such as chronic alcohol consumption, dietary habits, gastrointestinal disorders, and surgical interventions have been identified as primary contributors [[52], [53], [54], [55]], as they commonly impair intestinal thiamine absorption. Additionally, some cases of thiamine deficiency are associated with reduced enzymatic activity or defects in TPK1, which diminishes the conversion of thiamine to its active form, TPP [56,57]. Our findings indicate that CQ primarily reduces intracellular TPP concentrations without affecting thiamine levels, thereby directing attention toward the thiamine-to-TPP conversion process rather than thiamine uptake. Furthermore, our data demonstrate that CQ does not directly inhibit TPK1 enzymatic activity; instead, it suppresses TPK1 function indirectly by inducing the formation of thiamine oxidation products, such as thiochrome and thiamine disulfide.

This suggests that the deficiency of TPP may be associated with intracellular oxidative stress. Our study further demonstrated that CQ induces oxidative stress, which, in turn, leads to increased oxidative products of intracellular thiamine and TPP. Current research generally identifies mitochondria as the primary site of free radical generation within cells [58,59]. TPP is transported into mitochondria via the SLC25A19 transporter to exert its biological functions [60,61], which may explain why TPP undergoes greater oxidation than thiamine. Moreover, the oxidative products generated during this process inhibit TPK1, thereby exacerbating TPP deficiency. Our data shows that CQ administration did not significantly affect TPP levels in the brain tissue of wild-type mice, nor did it reduce thiamine levels (Figs. S8–9). Compared to the induction of TPP deficiency by CQ in the brain tissue of AD model mice, the effects of CQ in wild-type mice may suggest that organisms experiencing more severe oxidative stress are more susceptible to CQ-induced TPP deficiency. This segment of the study underscores the impact of oxidative stress on TPP metabolism and suggests that supplementation with thiamine or antioxidants could be beneficial in treating diseases associated with oxidative stress, such as Alzheimer's disease, Parkinson's syndrome, and Huntington's disease. Notably, investigations involving Alzheimer's patients have also reported a reduction in TPP levels in some cases [62]. Research has demonstrated that oral administration of benfotiamine in two clinical studies involving patients with Alzheimer's disease resulted in significant improvements in Mini-Mental State Examination (MMSE) scores and cognitive function [63]. Additionally, oral NAC has been shown to exert notable effects in animal models, including enhancement of passive avoidance memory in mice subjected to hippocampal injection of Aβ1-42 and in APP/PS1 transgenic mice, amelioration of contextual fear memory deficits induced by social isolation, and improvement of long-term hippocampal fear conditioning [64]. Our study may partially elucidate the alterations in thiamine metabolism observed in patients with Alzheimer's disease, as well as the beneficial effects of thiamine or antioxidant-based therapies.

Several studies have reported CQ's toxicity on energy metabolism [29,65]; however, these reports have primarily demonstrated CQ's inhibitory effect on intracellular ATP levels without providing an in-depth investigation of the underlying mechanisms. In contrast, our research offers a more detailed characterization of the energy metabolic disturbances induced by CQ and proposes TPP deficiency as a mechanistic explanation for these effects. Our findings demonstrate that CQ treatment reduces oxidative phosphorylation and concomitantly increases glycolytic activity. They are attributed to the impairment of TPP-dependent enzymes, such as pyruvate dehydrogenase, as evidenced by the accumulation of its substrate, pyruvate, which reduces TCA cycle flux. Consequently, to compensate for the diminished aerobic respiration, the organism upregulates glycolysis.

Our study highlights that TPP functions not only as a coenzyme for enzymes involved in cellular energy metabolism but also serves as a buffer, mitigating oxidative stress. Due to its multifaceted roles, TPP links oxidative stress responses with the reprogramming of energy metabolism. Thiamine plays a crucial role in maintaining the structural integrity and functional activity of cerebellar cells [66,67]. Thiamine depletion results in damage to the thalamus, which subsequently causes significant pathological changes in the cortex, stem cells, cerebellar area, and hippocampus, leading to cognitive and motor impairments [66,67]. Thiamine deficiency adversely affects microglial cells in the brain by impairing the regulation of peroxidases, thereby increasing oxidative stress within the nervous system and neural tissues of affected animals [68,69]. Furthermore, our findings suggest that tissues and cells with high metabolic activity, such as those in the nervous system, may be more susceptible to CQ's effects, particularly elucidating CQ's involvement in the pathogenesis of SMON. We explored combination drug regimens (CQ plus NAC or CQ plus TPP) that do not compromise CQ's ability to clear amyloid plaques while effectively reducing CQ-induced neurotoxicity in a mouse AD model. The TPP dosage used in this study (150 mg/kg/day) is still lower than that used in some studies on the neuroprotective effects of thiamine-class drugs in mouse models (>200 mg/kg/day) [12,13]. When converted to the equivalent human dose (∼12 mg/kg/day) according to FDA guidelines, it is also lower than some clinical doses (>75 mg/kg/day) [15,16], indicating that our regimen has translational potential. In the future, integrating multi-omics techniques is expected to strengthen current findings and clarify the mode of action of CQ in both in vitro and in vivo studies. Further research should focus on modifying CQ to minimize oxidative stress-induced cellular damage while preserving its therapeutic efficacy.

## 5 Conclusions

This study investigates the effects of CQ on thiamine and its derivatives, as well as the mechanisms underlying these effects. The findings indicate that CQ elevates intracellular oxidative stress, leading to the oxidation of TPP and its subsequent loss of functional activity. This process leads to mitochondrial dysfunction and metabolic reprogramming. The TPP deficiency and mitochondrial dysfunction induced by CQ can be mitigated by antioxidant administration. In a murine model of AD, oral CQ administration was found to concurrently decrease serum levels of both thiamine and TPP. However, antioxidant supplementation restored serum thiamine levels. These findings underscore the significance of thiamine and its derivatives in regulating the equilibrium between antioxidants and oxidants during periods of oxidative stress. The transient depletion of thiamine pyrophosphate during oxidative stress provides a novel perspective on the cellular energy metabolism disturbances and neurotoxicity associated with CQ. Taken together, the combination of CQ with other drugs, such as thiamine or TPP, represents a promising therapeutic strategy for neurodegeneration-related diseases.

## Animal experiments

All animal experiments were approved by the ECUST Animal Management and Ethics Committee (Approval No: ECUST-2025-016).

## CRediT authorship contribution statement

**Zheyu Fan:** Investigation, Methodology, Visualization, Writing – original draft, Writing – review & editing. **Xianghui Yan:** Investigation, Methodology. **Qiaoqiao Zheng:** Investigation, Methodology. **Ziting Zhu:** Investigation, Methodology. **Boyi Song:** Investigation, Methodology. **Xiao Feng:** Investigation, Methodology. **Siyu Pan:** Investigation, Methodology. **Chunyi Lv:** Investigation, Methodology. **Ping Shi:** Funding acquisition, Writing – review & editing.

## Declaration of competing interest

The authors declare that they have no known competing financial interests or personal relationships that could have appeared to influence the work reported in this paper.

## Data Availability

Data will be made available on request.

## Abbreviations

ASC: ascorbic acid
Aβ42: amyloid beta 42
CAT: catalase
CQ: Clioquinol
CRT: calreticulin
GSH: glutathione
HPLC: high-performance liquid chromatography
MDA: malondialdehyde
MMP: mitochondrial membrane potential
NAC: N-acetylcysteine
ROS: reactive oxygen species
SMON: subacute myelo-optic neuropathy
SOD: superoxide dismutase
TPK1: thiamine pyrophosphokinase 1
TPP: thiamine pyrophosphate
UPR: unfolded protein response
