# Supplementary material for: Clioquinol inactivates thiamine pyrophosphate by increasing cellular oxidative stress
Source: Redox Biol. 2026 Jun 17;95:104258. doi: 10.1016/j.redox.2026.104258 (PMC13320446; doi:10.1016/j.redox.2026.104258)
Supplement: Multimedia component 1 [file mmc1.docx]

**Supplemental Information for**

**Clioquinol inactivates thiamine pyrophosphate by increasing cellular oxidative stress**

**Files:**

Supplemental Figures 1-11 and Table 1


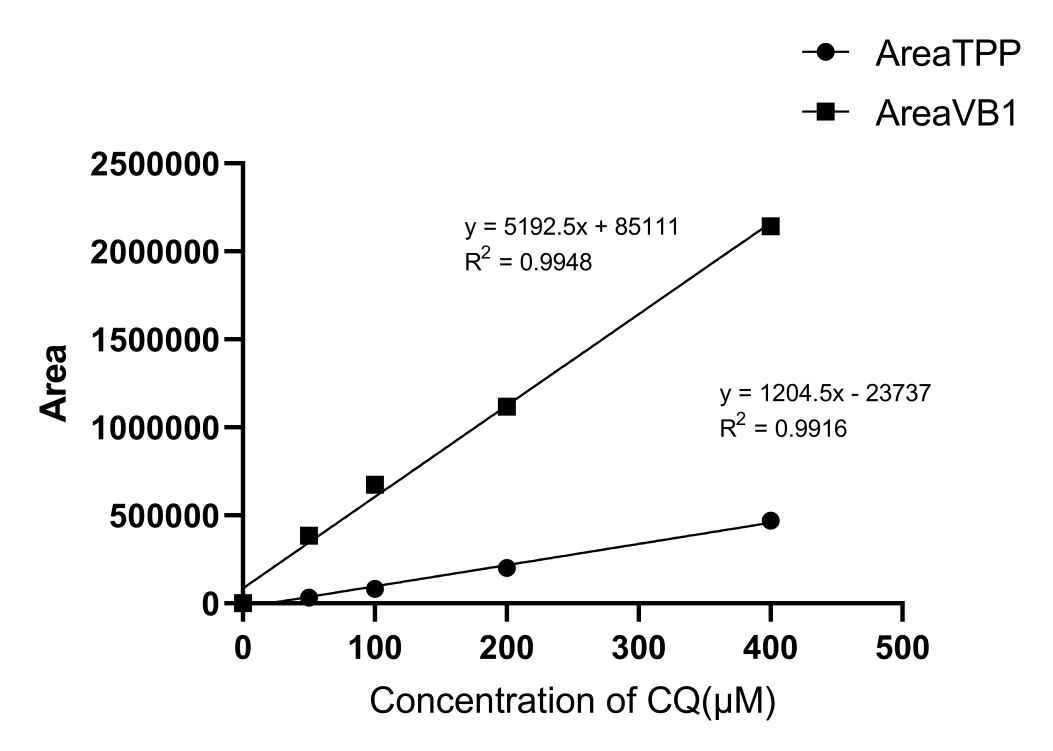


**Supplemental Fig. 1 Standard calibration curves for thiamine and TPP determined by HPLC method.**


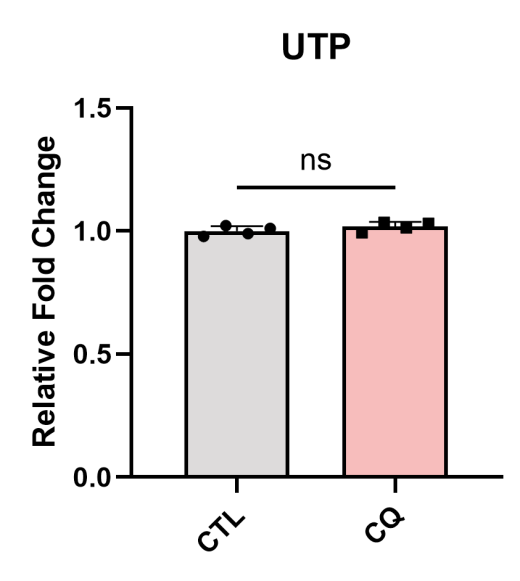


**Supplemental Fig. 2 The effect of CQ treatment for 24 hours on intracellular UTP levels.**


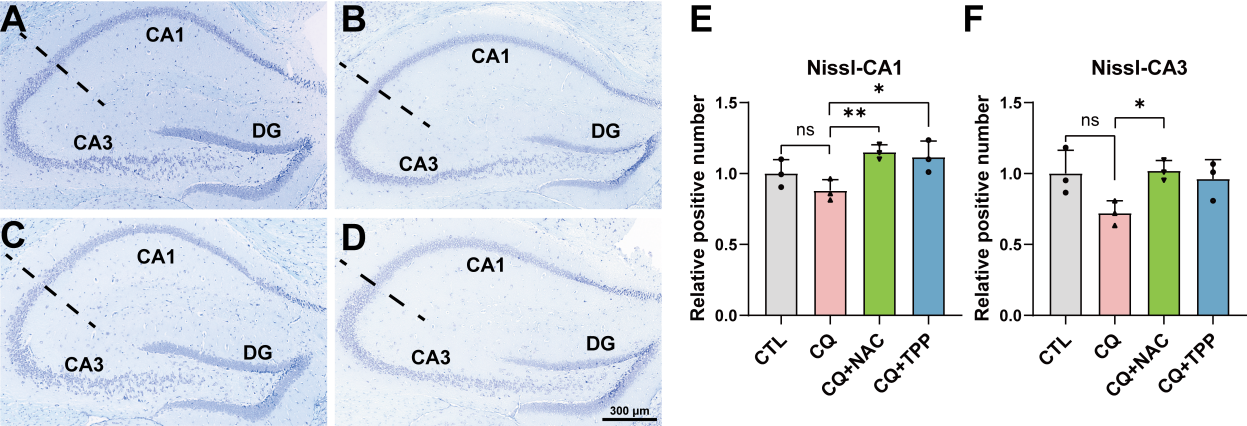


**Supplemental Fig. 3 Nissl staining to assess neuronal damage in AD model mice.** (A-D) Nissl staining images of AD model mice brain tissue in CTL (A) and CQ (B), CQ+NAC (C), and CQ+TPP (D) groups. (E-F) The bar chart shows the relative Nissl staining positive cell number of AD model mice hippocampus ca1 (E) and CA3 (F) in CTL and CQ, CQ+NAC, and CQ+TPP groups. Data were presented as mean±SE. *, P < 0.05; **, P < 0.01.


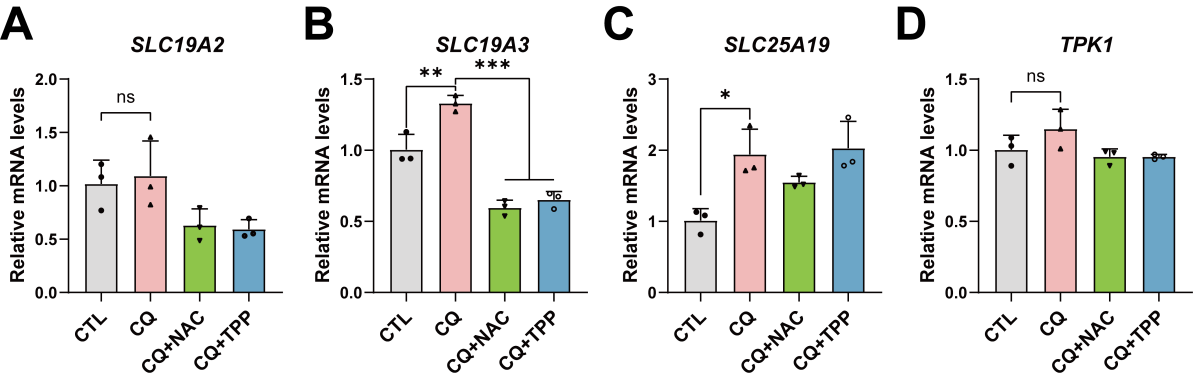


**Supplemental Fig. 4 mRNA expression levels of *SLC19A2, SLC19A3, SLC25A19* and *TPK1* in brain tissues of AD model mice.** (A) *SLC19A2****.*** (B) *SLC19A3****.*** (C) *SLC25A19****.*** (D) *TPK1****.*** Data (n=3) were presented as mean ± SE. *, P < 0.05; **, P < 0.01; ***, P < 0.001.


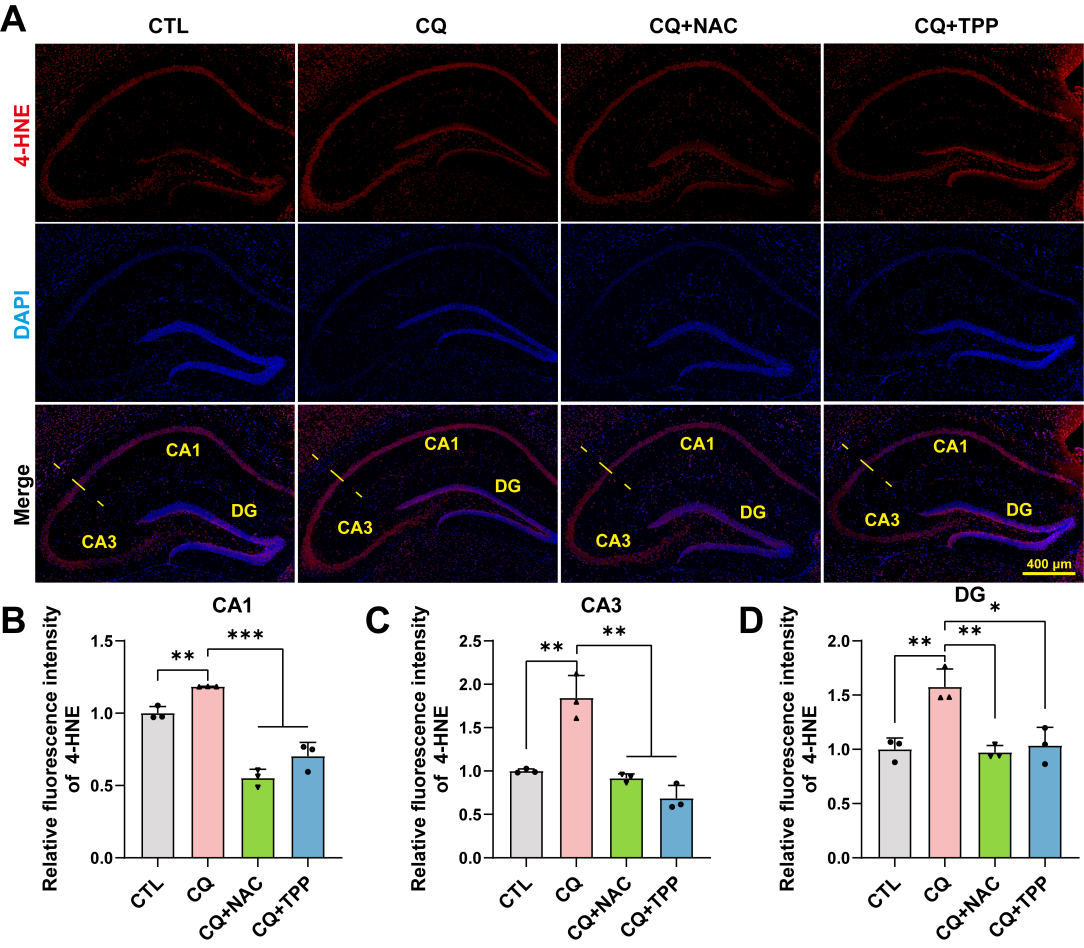


**Supplemental Fig. 5 Oxidative stress conditions in brain tissues of AD model mice.** (A) 4-HNE immunofluorescence images. (B-D) Bar graph showing the 4-HNE relative fluorescence intensity in the CA1 (B), CA3 (C), and DG (D) regions. Data (n=3) were presented as mean ± SE. *, P < 0.05; **, P < 0.01; ***, P < 0.001.


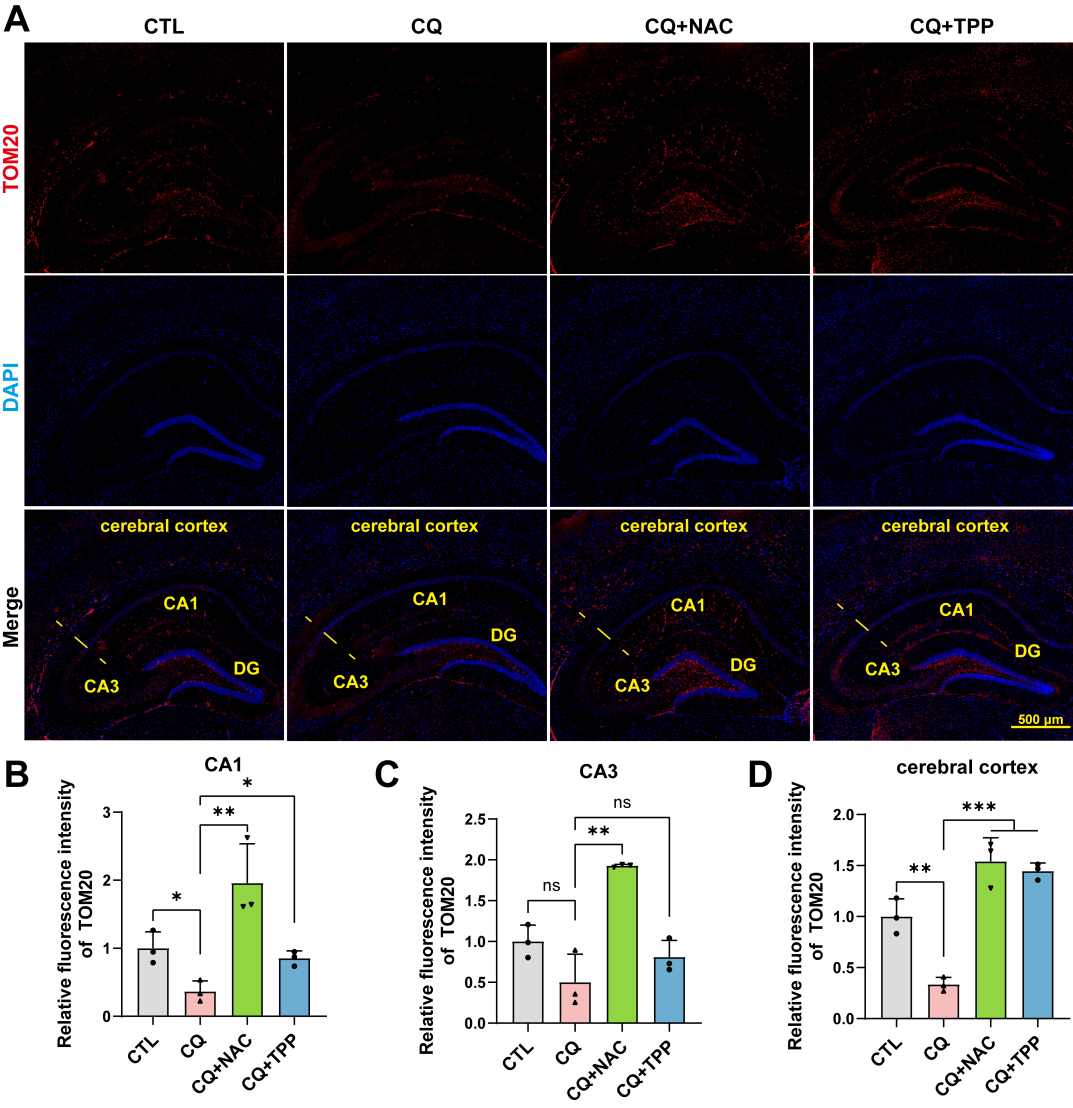


**Supplemental Fig. 6 Immunofluorescence results of TOM20 in brain tissue of AD model mice.** (A) TOM20 immunofluorescence images. (B-D) Bar graph showing the TOM20 relative fluorescence intensity in the CA1 (B), CA3 (C), and cerebral cortex (D) regions. Data (n=3) were presented as mean ± SE. *, P < 0.05; **, P < 0.01; ***, P < 0.001.


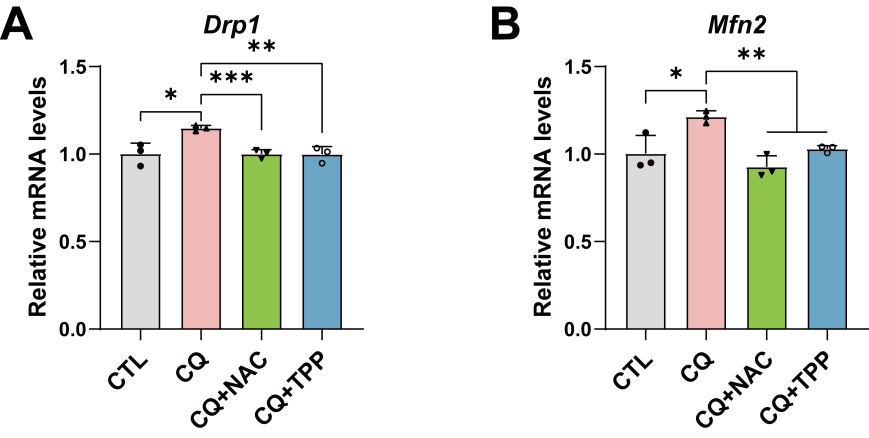


**Supplemental Fig. 7 mRNA expression levels of *Drp1* and *Mfn2* in brain tissues of AD model mice.** (A) *Drp1****.*** (B) *Mfn2****.*** Data (n=3) were presented as mean ± SE. *, P < 0.05; **, P < 0.01; ***, P < 0.001.


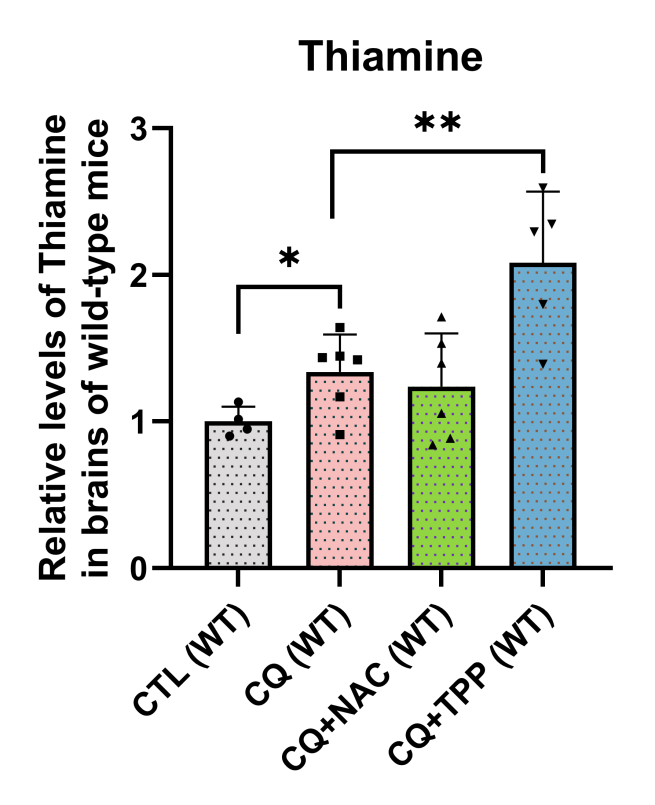


**Supplemental Fig. 8 Thiamine levels in the brain tissue of wild-type mice.** Data were presented as mean ± SE. *, P < 0.05; **, P < 0.01


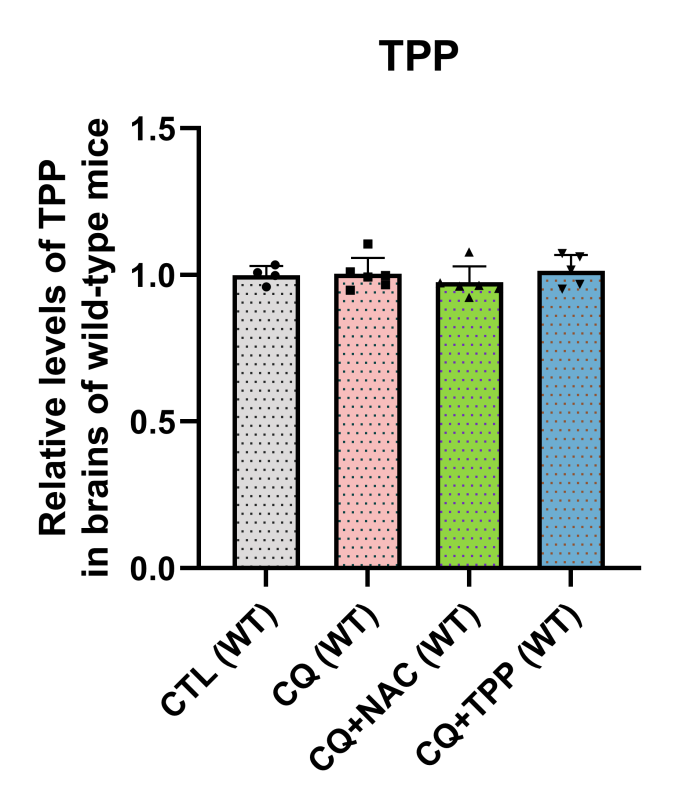


**Supplemental Fig. 9 TPP levels in the brain tissue of wild-type mice.** Data were presented as mean ± SE.


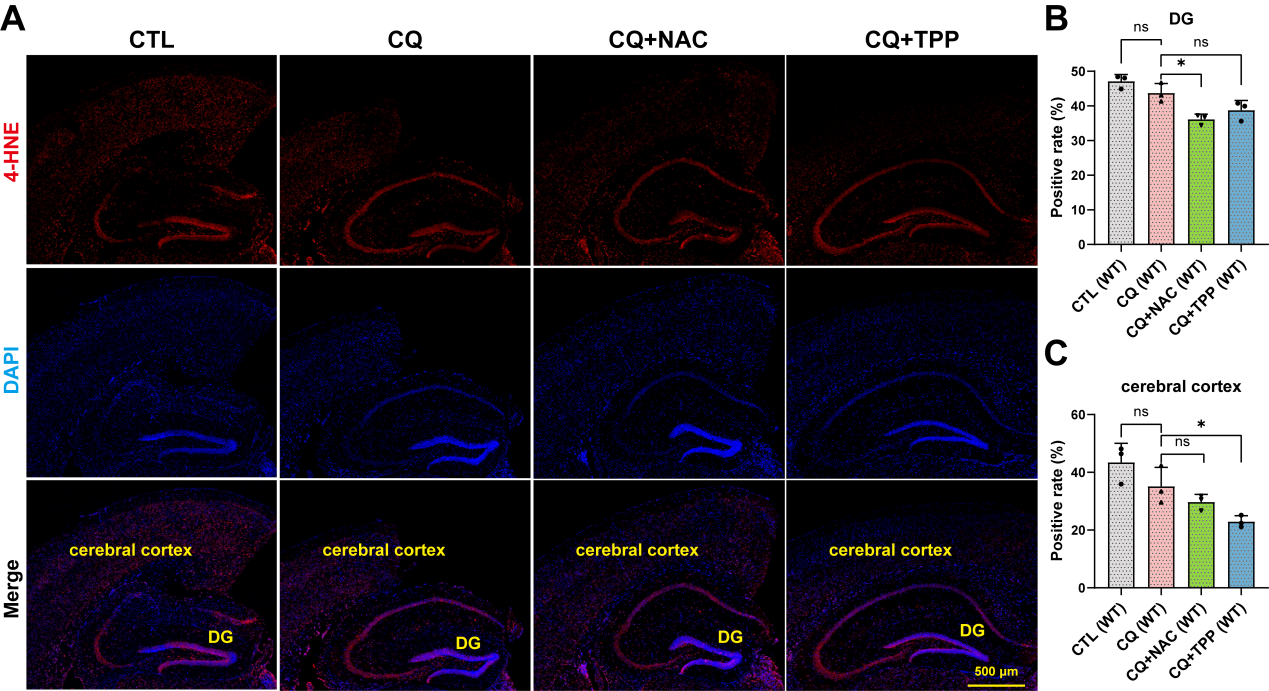


**Supplemental Fig. 10 Oxidative stress conditions in brain tissues of wild type mice.** (A) 4-HNE immunofluorescence images. (B-C) Bar graph showing the 4-HNE relative fluorescence intensity in the cerebral cortex (B) and DG (C). Data (n=3) were presented as mean ± SE.


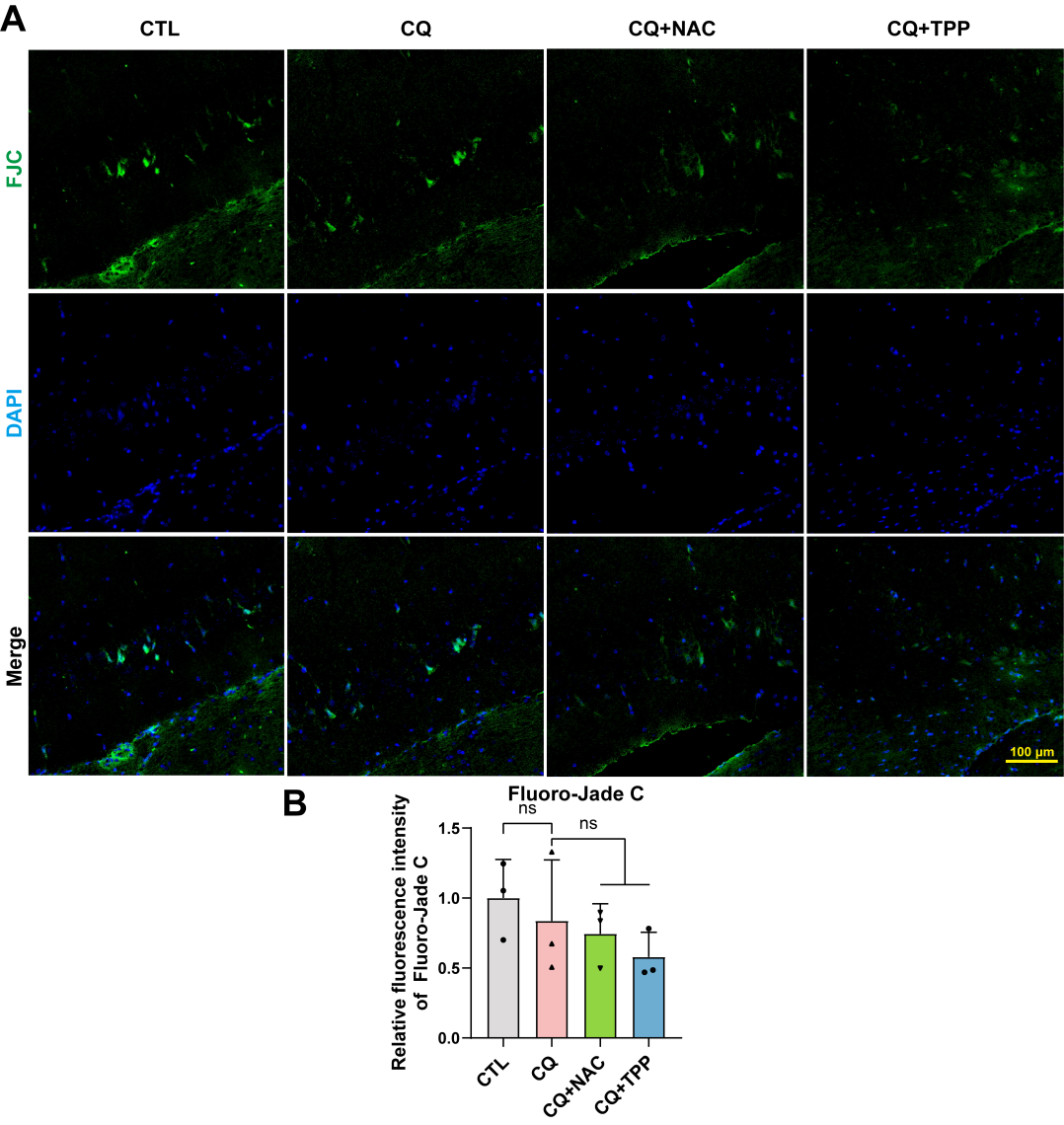


**Supplemental Fig. 11 Fluoro-Jade C staining results of brain tissue in AD model mice.** (A) Fluoro-Jade C staining images. (B) The bar graph shows the relative fluorescence intensity of FJC in the regions where positive cells are located. Data (n=3) were presented as mean ± SE.

**Supplemental Table 1 Primer Table**

| Genes | Species | Position | Sequences |
| --- | --- | --- | --- |
| *18S* | Human | Forward | CGCCTACCACATCCAAGGAAG |
|  |  | Reverse | AGCTGGAATTACCGCGGCT |
| *HPRT* | Human | Forward | AGACTTTGCTTTCCTTGGTCA |
|  |  | Reverse | AGGCTTTGTATTTTGCTTTTC |
| *SLC19A2* | Human | Forward | TTGCCACAGACTACCTCCGT |
|  |  | Reverse | GCACTTCGACAGTAACTTGTGA |
| *SLC19A3* | Human | Forward | CTGGCTCTGGTGGTCTTCTC |
|  |  | Reverse | AGGCATAGCGTTCCACATTC |
| *TPK1* | Human | Forward | CCTGAATTCATCAATGGAGACTTTG |
|  |  | Reverse | AGCAAGCACATCATTTGTGAGG |
| *SLC25A19* | Human | Forward | TCCAGGCCTCTAGGCAGATT |
|  |  | Reverse | CCAGCGGATATGTCAGGGTC |
| *SLC19A2* | Mouse | Forward | TGAACGGCCTCAAGGAGGAG |
|  |  | Reverse | GGATGCATAGCACACCCAGA |
| *SLC19A3* | Mouse | Forward | GAGATCGCCGACCCACATAG |
|  |  | Reverse | AGTTGCTCGGTGGAGTTCTG |
| *SLC25A19* | Mouse | Forward | GGCTTGGAAGCGGAAGACTA |
|  |  | Reverse | CGTATGGCCTCTCGCAGATT |
| *TPK1* | Mouse | Forward | TCAGCCTTTGGATGCACGAT |
|  |  | Reverse | CAGGTAAGGGAAGGCACAGG |
| *Actb* | Mouse | Forward | GATATCGCTGCGCTGGTCG |
|  |  | Reverse | CATTCCCACCATCACACCCT |
| *Mfn2* | Mouse | Forward | TCCTCTCCCTCTGACACCTG |
|  |  | Reverse | TCCTCCGACCACAAGAATGC |
| *Drp1* | Mouse | Forward | TGCAGGACGTCTTCAACACA |
|  |  | Reverse | GACCACACCAGTTCCTCTGG |
